# Potential Health Risks Associated with Sports Supplementation Use in Athletes

**DOI:** 10.3390/nu18152490

**Published:** 2026-08-02

**Authors:** Jiaqin Cai, Tutu Wang, Shunchang Li

**Affiliations:** Sports Medicine Key Laboratory of Sichuan Province, Institute of Sports Medicine and Health, Chengdu Sport University, Chengdu 641418, China; caijiaqin2002@gmail.com (J.C.); tutuwang980306@gmail.com (T.W.)

**Keywords:** sports supplements, athletes, endocrine homeostasis disruption, cardiovascular adverse effects, gastrointestinal symptoms, hepatotoxicity, kidney injury

## Abstract

As modern competitive sports continue to evolve, athletes are under increasing pressure to optimize both performance and recovery. Sports supplements have therefore become widely used among athletes because of their purported benefits in enhancing athletic performance, delaying fatigue, and accelerating physiological recovery. However, misuse, excessive intake, and other inappropriate patterns of supplement use remain common in some athletes and may impair performance, compromise health, and increase the risk of anti-doping rule violations. Although existing research has largely focused on the ergogenic benefits of sports supplements, their potential health risks in athletes remain insufficiently characterized. This review summarizes the potential adverse effects of sports supplements on the endocrine, cardiovascular, and gastrointestinal systems, with an emphasis on the underlying pathological mechanisms, thereby providing a theoretical basis for the evidence-based selection and responsible use of sports supplements in athletic populations.

## 1. Introduction

As the standard of competitive sport continues to rise, athletes are exposed to greater physical demands during training and competition, more stringent recovery requirements, and increasingly congested competition schedules [1,2]. To enhance athletic performance, delay fatigue, and accelerate physiological recovery, sports supplements have been widely incorporated into athletes’ daily training and competition routines and have gradually become an important component of sports nutrition support [3]. Athletes represent the primary users of sports supplements, with prevalence rates substantially higher than those observed in the general population, typically ranging from 40% to 80%; among elite athletes, supplement use may even exceed 90% [4,5,6]. These findings indicate that the use of sports supplements has become an integral part of routine athletic training.

Multiple studies have demonstrated that athletes’ motivations for using sports supplements are multifaceted, encompassing various objectives such as improving health status, enhancing athletic performance, accelerating post-exercise recovery, and modulating immune function [7,8,9,10]. Among these, health improvement and performance enhancement represent the primary motivations reported across most studies; however, their relative importance is influenced by multiple factors, including geographic region, competitive level, and sex, resulting in substantial heterogeneity across athlete populations [11,12,13,14]. Notably, athletes with disabilities exhibit distinct physiological characteristics and nutritional requirements due to variations in disability types and functional status, leading to considerable inter-individual heterogeneity in their needs for sports supplementation [15,16,17]. A study by Madden et al. in elite Paralympic athletes revealed that the primary motivations for sports supplement use in this population were health maintenance, energy provision, and medical-related needs. Although the overall pattern of motivations was comparable to that observed in able-bodied athletes, medical considerations exerted a more prominent influence [18]. Furthermore, supplement use among athletes is not solely driven by individual decision-making; social and environmental factors, including recommendations from coaches and behaviors modeled by peers, also play important roles. These findings suggest that sports supplement use represents the interplay between individual needs and social influences [19]. Collectively, these diverse motivations highlight that sports supplements have become deeply integrated into the contemporary training and competitive practices of athletes.

However, although sports supplements may offer potential benefits for improving performance and promoting recovery, their safety remains insufficiently supported by robust evidence [20]. Previous studies have shown that long-term or high-dose use of certain sports supplements may exert adverse effects on multiple organ systems, particularly key target organs such as the cardiovascular system, liver, and kidneys [21]. For the purposes of this review, long-term supplementation was operationally defined as continuous or repeated intake for at least 8 weeks, or supplementation sustained over a complete training cycle or competitive season [22]. High-dose supplementation was defined as an intake exceeding one or more established reference thresholds, including recommended daily intake levels, tolerable upper intake levels (ULs), evidence-based ergogenic doses, or manufacturer-recommended dosages [23,24]. Such damage may not only compromise athletic performance but also negatively affect athletes’ long-term health and quality of life [21]. Nevertheless, most athletes still have relatively limited awareness of the potential health risks associated with sports supplements. At the same time, misleading commercial claims and marketing messages have contributed to the increasingly common irrational use and even misuse of these products [6,25]. In this context, for athletes who use sports supplements over prolonged periods, insufficient understanding of the potential harms associated with inappropriate use may prevent them from achieving the intended benefits and may instead adversely affect their health [26]. For example, long-term or high-dose intake of exogenous antioxidants such as vitamin C or vitamin E may disrupt the dynamic balance between free radicals and endogenous antioxidant defense systems, thereby promoting lipid peroxidation and inflammatory responses and exacerbating oxidative stress-related injury. In addition, excessive antioxidant supplementation may inhibit the activation of reactive oxygen species (ROS)-mediated exercise-adaptive signaling pathways, attenuating exercise-induced adaptive responses and altering the expression of adaptation-related genes and protein synthesis, ultimately impairing skeletal muscle function and cardiovascular health [27]. Therefore, the use of sports supplements should not be considered solely from the perspective of potential benefits; rather, their safety concerns warrant more systematic attention.

This review was based on a systematic search and screening of the English-language literature in PubMed, Web of Science, and Scopus. The search primarily covered studies published over the past two decades, from 2006 to 2026. In addition, earlier studies that were highly relevant to the topic of this review and had substantial mechanistic, clinical cautionary, or foundational value were also included to ensure the completeness of the evidence chain and the continuity of the discussion.

The search terms were structured around three core themes: athletes, sports supplements, and potential health risks. The main search terms included: “athletes” OR “sports” OR “exercise”; “sports supplements” OR “dietary supplements” OR “ergogenic aids” OR “performance-enhancing supplements”; and “injury” OR “adverse effects” OR “toxicity” OR “health risks” OR “organ damage” OR “endocrine homeostasis disruption” OR “cardiovascular adverse effects” OR “gastrointestinal symptoms” OR “hepatotoxicity” OR “kidney injury.” These terms were combined using Boolean operators according to the search rules of each database.

The inclusion criteria were as follows: (1) studies directly related to athletes, sports supplements, and their potential health risks; (2) studies with sufficient experimental data, clinical evidence, or epidemiological information to support their main conclusions; (3) studies published in peer-reviewed academic journals; and (4) studies including experimental studies, animal studies, observational studies, clinical trials, case reports, systematic reviews, meta-analyses, and foundational narrative reviews. The exclusion criteria were as follows: (1) studies with methodological flaws or insufficient data support; (2) studies not directly relevant to the topic of this review; (3) studies in which the study population, intervention, or outcome measures did not align with the focus of this review; and (4) studies that were not peer reviewed, had unclear sources, or consisted only of conference abstracts, news commentaries, or nonacademic materials.

The initial search identified 407 records. After removal of duplicates, 357 records remained for title and abstract screening. Following this preliminary screening, 300 articles were selected for full-text assessment, and 234 studies were ultimately included for evidence synthesis in this review. For supplement categories with a relatively robust evidence base, we prioritized studies with rigorous designs, larger sample sizes, higher levels of evidence, or more recent publication dates. For supplements with limited evidence but important safety implications, case reports, animal studies, and mechanistic studies were included as supplementary evidence. Because this review is a narrative review rather than a systematic review or scoping review in the strict sense, no formal risk-of-bias assessment or evidence-quality grading was performed. Nevertheless, to improve the transparency and reproducibility of the literature selection and reporting process, we referred to the relevant reporting principles of PRISMA-ScR during the identification, screening, and inclusion of studies.

Although research on sports supplements has increased in recent years, existing studies have primarily focused on their beneficial effects in improving athletic performance and alleviating exercise-induced fatigue, whereas their potential harms to athlete health have not been systematically summarized or integrated. Accordingly, this review focuses on the potential adverse effects of sports supplements on athlete health and systematically summarizes their major risks and possible mechanisms, with the aim of providing a theoretical basis for the rational use of sports supplements among athletes.

## 2. Classification of Sports Supplements

Athletes’ motivations for using sports supplements are multidimensional, which is reflected in the considerable diversity of supplement categories and functional claims [28]. Therefore, before discussing their potential harms, it is necessary to first summarize the current state of research in this field. At present, the ABCD classification system proposed by the Australian Institute of Sport (AIS) is one of the most authoritative international frameworks for categorizing sports supplements. This system classifies supplements into four groups according to the strength and quality of evidence: Group A includes supplements with strong scientific evidence supporting their use in specific contexts; Group B includes supplements that warrant further investigation but currently have insufficient evidence; Group C includes supplements for which meaningful evidence of efficacy is lacking; and Group D includes supplements that are prohibited or associated with a high risk of contamination [29]. Based on this framework, the present review further categorizes sports supplements into two broad groups—classical sports supplements and emerging sports supplements—and summarizes the current research status of each category (Table 1).

### 2.1. Classical Sports Supplements

Classical sports supplements generally refer to supplements that have been extensively studied over time, have relatively well-defined mechanisms of action, and are widely used in the field of sports nutrition. Most of these supplements are classified by the AIS as Group A or Group B. Based on their primary compositional characteristics, this review further divides classical sports supplements into proteins and amino acid derivatives; carbohydrates and electrolytes; vitamins, minerals, and bioactive phytochemicals; hormones and androgenic anabolic agents; and alkaloids and nitrophenol compounds.

#### 2.1.1. Proteins and Amino Acid Derivatives

Sports supplements based on proteins and amino acid derivatives represent one of the most extensively studied and widely used nutritional strategies in sports nutrition. This category primarily includes protein supplements such as whey protein and casein and amino acid derivative supplements, with creatine being the most representative example [30,31]. The core functions of these supplements include attenuating exercise-induced muscle damage, promoting post-exercise muscle protein synthesis, and accelerating recovery from exercise-induced fatigue [30,31,32].

Among protein supplements, whey protein is a protein fraction derived from by-products of dairy processing. Owing to its rapid digestion and absorption kinetics and high leucine content, whey protein is widely regarded as one of the most effective protein sources for stimulating post-exercise muscle protein synthesis [33]. Casein, the most abundant protein in mammalian milk, exists as a micellar phosphoprotein. Its slow and sustained amino acid release profile makes it particularly suitable for protein supplementation during prolonged fasting windows, such as before overnight sleep, with the aim of sustaining muscle protein synthesis following endurance exercise [34,35].

In addition to protein, supplements such as whey protein and casein, an amino acid derivative, have also received considerable attention in sports nutrition [36]. Studies have shown that creatine supplementation can improve strength performance and power output during repeated high-intensity exercise, making it particularly relevant for sports that require explosive power and repeated sprint ability [37]. At the same time, available evidence indicates that creatine use in healthy athletes is generally well tolerated and has a favorable safety profile [38]. However, creatine supplementation is commonly accompanied by increases in body mass, which may limit its practical application in certain weight-sensitive sports [39].

#### 2.1.2. Carbohydrates and Electrolytes

Carbohydrate supplements represented by maltodextrin, glucose, and fructose, together with sodium bicarbonate (NaHCO_3_)-based electrolyte supplements, are important components of sports nutrition intervention strategies. In competitive sports, these two types of supplements are often used in combination to synergistically support energy provision and fluid–electrolyte balance [40,41].

Among carbohydrate supplements, maltodextrin is one of the most widely used forms. Its primary role is to provide a rapidly available source of exogenous carbohydrate during exercise, thereby helping maintain blood glucose homeostasis, reduce endogenous glycogen depletion, and delay the onset of fatigue [42]. In addition, because the osmolality of maltodextrin solutions is markedly lower than that of isoenergetic monosaccharide solutions, maltodextrin offers advantages in promoting gastric emptying and improving gastrointestinal tolerance. Consequently, it is widely incorporated into the formulations of sports drinks and energy gels [43]. However, supplementation with a single carbohydrate source is limited by the saturation of a single intestinal transporter, making it difficult to further increase the oxidation rate of exogenous carbohydrates [44]. On this basis, combined carbohydraminte supplementation strategies involving maltodextrin or glucose with fructose have received increasing attention in recent years. By simultaneously utilizing different intestinal sugar transporters, this strategy can support higher rates of exogenous carbohydrate oxidation and has been shown to improve endurance performance during exercise lasting 2.5 h or longer. It has therefore become a mainstream carbohydrate supplementation approach for prolonged endurance events [45,46].

Among electrolyte supplements, NaHCO_3_ is an inorganic alkaline salt that acts as an extracellular buffering agent, exerting dual effects by contributing to electrolyte replenishment and the regulation of acid–base balance [47]. Following oral ingestion, NaHCO_3_ can significantly increase plasma bicarbonate concentration and blood pH, thereby attenuating exercise-induced muscle acidosis [47]. It is primarily used in high-intensity exercise events that rely predominantly on glycolytic energy metabolism, with the aim of improving anaerobic performance during efforts lasting approximately 1–7 min [41].

#### 2.1.3. Vitamins, Minerals, and Bioactive Phytochemicals

Among vitamin, mineral, and bioactive phytochemical supplements, vitamin D, vitamin C, iron, and epigallocatechin-3-gallate (EGCG) are among the most extensively studied and widely used supplements in sports nutrition [48,49,50].

Among vitamin supplements, vitamin D is currently one of the most widely investigated exercise-related vitamins [51]. Multiple epidemiological studies have shown that vitamin D deficiency is common among athletes, particularly those who train at high latitudes, participate primarily in indoor sports, or have limited sunlight exposure [52]. Evidence indicates that targeted vitamin D supplementation in athletes with deficiency may help improve muscle strength, reduce the risk of stress fractures, and enhance upper respiratory immune defense [52]. However, for athletes with sufficient vitamin D status, whether additional vitamin D supplementation can improve athletic performance remains inconsistent across studies [52]. In addition, vitamin C, as a classical antioxidant vitamin, has long been used to counteract exercise-induced oxidative stress [53,54]. However, subsequent research by Li et al. found that high-dose supplementation with exogenous antioxidant vitamins may excessively scavenge ROS generated during exercise, thereby weakening the crucial role of reactive oxygen species as signaling molecules in mediating training adaptation [27].

Among mineral supplements, iron is one of the trace elements most closely associated with athletic performance and supported by a substantial body of evidence [55,56]. Athletes, especially female athletes, are at high risk of iron deficiency. This phenomenon is related to menstrual blood loss and multiple forms of exercise-associated iron loss, which may lead to iron-deficiency anemia and consequently impair athletic performance [57,58]. Current evidence suggests that oral iron supplementation in iron-deficient athletes can improve iron stores and hematological iron-status markers [56]. Notably, the potential benefits of iron supplementation are mainly limited to targeted use in athletes with iron deficiency; for athletes with adequate iron stores, the effectiveness of routine iron supplementation remains to be further clarified [59].

Among bioactive phytochemical supplements, EGCG is the most abundant and biologically active catechin polyphenol in green tea, accounting for approximately 50–80% of total tea catechins. It is considered a key effector through which tea polyphenols exert their biological functions [60]. Owing to its pronounced antioxidant and anti-inflammatory properties, EGCG has been widely used to attenuate high-intensity exercise-induced oxidative stress and systemic inflammatory responses, thereby accelerating post-exercise recovery [50]. In addition, EGCG promotes fat oxidation and enhances exercise-induced thermogenesis, and is therefore used by some bodybuilders as an effective pre-competition fat-loss strategy [61,62].

#### 2.1.4. Hormones and Androgenic Anabolic Agents

Among hormone-related and androgenic anabolic supplements, triiodothyronine (T_3_), anabolic–androgenic steroids (AAS), and selective androgen receptor modulators (SARMs) are widely used by athletes to manipulate body composition during fat-loss and muscle-gain phases [63,64,65].

Among hormone-based supplements, T_3_ is the most biologically active form of thyroid hormone. Endogenous T_3_ is primarily generated in peripheral tissues through the 5′-deiodination of thyroxine (T_4_), catalyzed by type I and type II iodothyronine deiodinases [66]. Exogenous T_3_, as a synthetic pharmacological form of this hormone, is widely used during contest preparation in bodybuilders to accelerate fat loss by markedly increasing basal metabolic rate, promoting fatty acid oxidation, and thereby elevating total energy expenditure [63].

Among androgenic anabolic supplements, AAS are steroidal compounds derived from the endogenous testosterone scaffold through structural modification. Their oral bioavailability can be increased by C17α-alkylation, whereas esterification can prolong their duration of action after injection [67]. Supraphysiological doses of AAS can markedly increase the rate of net skeletal muscle protein synthesis, thereby enlarging muscle fiber cross-sectional area, which has made them one of the most widely used muscle-building supplements among strength athletes and bodybuilders [64]. Compared with AAS, SARMs are nonsteroidal ligands that exert muscle-selective anabolic effects, providing greater tissue selectivity while promoting muscle gain [65]. These compounds were originally developed as clinical interventions for sarcopenia and have now become a common alternative to AAS among strength athletes and bodybuilders [65].

#### 2.1.5. Alkaloids and Nitrophenol Compounds

Alkaloid supplements, such as caffeine and ephedrine, and nitrophenol supplements, exemplified by 2,4-dinitrophenol (2,4-DNP), are among the relatively well-studied sports supplements and are commonly used in athletic populations [68,69,70].

Among alkaloid supplements, caffeine is a methylxanthine alkaloid widely found in natural plant sources, including coffee beans, tea leaves, and cocoa [71]. Numerous studies have shown that caffeine effectively attenuates exercise-induced fatigue and significantly enhances performance across multiple exercise modalities. It is therefore one of the most evidence-based ergogenic supplements in sports nutrition and is used at a very high rate among competitive athletes across different sports [68,72]. Ephedrine is a sympathomimetic alkaloid derived from the stems and branches of *Ephedra* species. Owing to its central nervous system-stimulatory activity and peripheral sympathomimetic effects, ephedrine was once widely used in competitive sports to enhance power output during short-duration, high-intensity exercise and to prolong time to exhaustion [69,73]. In addition, its pronounced thermogenic and appetite-suppressing effects have made ephedrine a pre-competition rapid weight-loss strategy for athletes in weight-category sports, such as boxing and judo [69,74]. These effects are further potentiated when ephedrine is co-administered with caffeine [75].

Among nitrophenol supplements, 2,4-DNP is a synthetic mitochondrial uncoupler that was initially used primarily as an industrial dye, wood preservative, and herbicide [76]. In the early 1930s, 2,4-DNP was first introduced clinically as a weight-loss drug because it markedly increases basal metabolic rate, promotes fat oxidation, and thereby induces a pronounced thermogenic effect [77,78]. In contemporary competitive sports, bodybuilders use 2,4-DNP for body-weight management owing to its thermogenic properties [70].

### 2.2. Emerging Sports Supplements

Emerging sports supplements generally refer to supplements that have gradually attracted attention and become increasingly used in recent years, while the related evidence base is still evolving. Because no universally accepted classification criteria have yet been established and different emerging supplements are difficult to incorporate into a unified classification system, the following section focuses on several emerging sports supplements that are currently widely used, including exogenous ketones, tart cherry supplements, curcumin, probiotics, and ashwagandha, and summarizes the current research status of each.

#### 2.2.1. Exogenous Ketones

Exogenous ketones refer to a class of supplements that rapidly increase circulating ketone body concentrations through the oral intake of ketone salts, ketone esters, or their precursor compounds [79]. Existing studies have shown that exogenous ketone ingestion can, to some extent, alter substrate utilization during exercise, primarily by reducing peripheral tissue reliance on glucose, inhibiting adipose tissue lipolysis, and decreasing skeletal muscle protein breakdown [79]. This metabolic shift suggests that exogenous ketones may have potential applications in optimizing energy utilization and supporting the maintenance of exercise capacity and post-exercise recovery under specific exercise conditions. However, although these mechanisms are theoretically plausible, current evidence remains insufficient to confirm their effectiveness in improving athletic performance or enhancing post-exercise recovery [79].

#### 2.2.2. Tart Cherry Supplements

Tart cherry supplements are usually derived from Montmorency tart cherries, with common forms including concentrate juice and freeze-dried powder [80]. As plant-derived sports supplements rich in anthocyanins and polyphenols, they have attracted attention in sports nutrition primarily for their potential role in promoting recovery after strenuous exercise [80,81]. Multiple randomized controlled trials have shown that tart cherry supplementation can alleviate post-exercise muscle soreness and reduce markers associated with muscle damage [81]. However, current evidence remains inconsistent regarding its regulatory effects on blood biomarkers such as creatine kinase and inflammatory cytokines, as well as its direct impact on athletic performance [81,82,83]. In addition, although tart cherry supplementation may accelerate post-exercise recovery through anti-inflammatory and antioxidant mechanisms, whether this effect attenuates long-term training adaptations mediated by ROS and inflammatory signaling remains to be further investigated [84].

#### 2.2.3. Curcumin

Curcumin is a natural yellow polyphenol derived from the rhizome of turmeric and possesses biological properties such as anti-inflammatory, antioxidant, and immunomodulatory effects [85]. Its application in sports nutrition is highly similar to that of tart cherry supplements, with a primary focus on alleviating post-exercise muscle soreness, reducing exercise-induced muscle damage, and promoting post-exercise recovery [86]. However, consistent evidence supporting a direct ergogenic effect of curcumin on athletic performance is still lacking [86]. Compared with tart cherry supplements, curcumin faces a more prominent pharmacokinetic limitation: its oral bioavailability is extremely low, as most of it is rapidly metabolized in the intestine and liver [87]. Although formulation optimization strategies, such as liposomal encapsulation and co-administration with piperine, have improved this limitation to some extent, the marked differences in bioavailability among commercial formulations pose challenges for cross-study comparability and clinical translation [87].

#### 2.2.4. Probiotics

In the field of sports nutrition, interest in probiotics is not directed primarily toward athletic performance itself, but rather toward their potential to indirectly influence overall health by modulating intestinal barrier function and immune homeostasis [88]. The 2019 position stand of the International Society of Sports Nutrition (ISSN) stated that specific probiotic strains may help reduce the risk of upper respiratory tract infections in athletes and improve exercise-induced gastrointestinal discomfort [89]. However, the effects of probiotics are highly strain-specific and are also influenced by multiple interacting factors, including supplementation dose, intervention duration, and the individual’s baseline gut microbiome composition. This substantial heterogeneity makes it difficult to establish a universally applicable supplementation strategy for athletes based on the current evidence [89].

#### 2.2.5. Ashwagandha

Ashwagandha, as an adaptogenic herb, has attracted increasing attention in sports nutrition in recent years because of its potential roles in stress resistance, recovery support, and modulation of athletic performance, and it is therefore regarded as an emerging sports supplement [90]. Existing studies suggest that ashwagandha supplementation may, to some extent, improve aerobic capacity, muscular strength performance, and post-exercise recovery in athletes, while also attenuating exercise-induced stress responses [90]. However, current evidence is still mainly derived from small-sample randomized controlled trials, and its effects may be jointly influenced by participant characteristics, training status, and supplementation dose [90,91]. Therefore, although ashwagandha shows potential application prospects in sports nutrition, its practical value as a sports supplement remains to be further clarified.

**Table 1:** Main Characteristics and Functions of Classical and Emerging Sports Supplements.

Category	Subcategory	Sports Supplement	AIS Classification	Level of Evidence (GRADE)	Main Characteristics	Main Functions	Main Adverse Effects	Regulatory Status	Applicable Athletic Populations	References
Classical sports supplements	Proteins and amino acid derivatives	Whey protein	Group A	High	Rapid digestion and absorption, high leucine content	Promote rapid post-exercise muscle protein synthesis	Mild GI discomfort	Allowed	Competitive athletes	[33]
		Casein	Group A	Moderate	Slow and sustained release of amino acids	Support sustained muscle protein synthesis during prolonged fasting periods	Mild bloating	Allowed	Endurance athletes	[35]
		Creatine	Group A	High	Amino acid derivatives	Improve strength performance and power output	Water-retention weight gain	Allowed	Athletes in high-intensity repeated-exercise sports	[36,37]
	Carbohydrates and electrolytes	Maltodextrin	Group A	High	Enhance gastric emptying and gastrointestinal tolerability	Maintain blood glucose homeostasis, reduce endogenous glycogen depletion, delay the onset of fatigue	GI distress	Allowed	Competitive athletes	[42,43]
		Co-ingestion of maltodextrin (or glucose) and fructose	Group A	High	Support higher rates of exogenous carbohydrate oxidation	Enhance exercise performance	GI distress	Allowed	Endurance athletes	[45,46]
		NaHCO_3_	Group A	High	Exogenous alkalinizing agents	Attenuate exercise-induce muscle acidosis	GI distress	Allowed	Athletes engaged in high-intensity sports primarily powered by glycolytic metabolism	[41,47]
	Vitamins, minerals, and bioactive phytochemicals	Vitamin D	Group A	Low	High prevalence of vitamin D deficiency among athletes	Improve muscle strength, reduce risk of stress fractures, enhance upper respiratory immune defense	Hypercalcemia	Allowed	Athletes with vitamin D deficiency	[52]
		Vitamin C	Group B	Low	An antioxidant vitamin	Protect against exercise-induce oxidative stress	Blunted training adaptations	Allowed	Competitive athletes	[53]
		Iron	Group A	Moderate	One of the trace elements most closely linked to exercise performance	Improve iron stores and hematological markers of iron status	GI intolerance	Allowed	Athletes with iron deficiency, particularly female athletes	[56,57]
		EGCG	Group B	Low	The most abundant and biologically active catechin polyphenol in green tea	Attenuate exercise-induce oxidative stress and systemic inflammation, enhance fat oxidation	Hepatotoxicity	Allowed	High-intensity sport athletes, Bodybuilders	[50,61,62]
	Hormones and androgenic anabolic agents	T_3_	-	Very Low	The most biologically active form of thyroid hormone	Increase basal metabolic rate, promote fatty acid oxidation	Thyrotoxicosis, arrhythmia, muscle wasting	Prohibited	Bodybuilders	[63,66]
		AAS	Group D	High	Steroidal compounds	Increase the rate of net skeletal muscle protein synthesis	cardiovascular disease, HPG-axis suppression, hepatotoxicity	Prohibited	Strength athletes, Bodybuilders	[64,67]
		SARMs	Group D	Low	Nonsteroidal ligands	Selectively increase the rate of net skeletal muscle protein synthesis	Testosterone suppression, hepatotoxicity	Prohibited	Strength athletes, Bodybuilders	[65]
	Alkaloids and nitrophenol compounds	Caffeine	Group A	High	Methylxanthine alkaloids	Attenuate exercise-induced fatigue and enhance exercise performance	Insomnia, tachycardia, anxiety	Allowed	Competitive athletes	[68,71,72]
		Ephedrine	Group D	Low	Sympathomimetic amine alkaloids	Increase power output, prolong time to exhaustion, promote weight loss	Hypertension, arrhythmia, stroke	Prohibited	Short-duration high-intensity sport athletes, Weight-class sport athletes	[69,74]
		2,4-DNP	-	Very Low	A synthetic mitochondrial uncoupler	Increase basal metabolic rate, promote fat oxidation	Hyperthermia, rhabdomyolysis	Prohibited	Bodybuilders	[70,76,77,78]
Emerging sports supplements		Exogenous ketones	Group B	Low	Can alter substrate utilization patterns during exercise	Optimize energy utilization, support the maintenance and recovery of exercise capacity	GI distress	Allowed	Competitive athletes	[79]
		Tart cherry supplements	Group B	Moderate	Anthocyanin- and polyphenol-rich	Attenuate post-exercise muscle soreness, reduce exercise-induced muscle damage, accelerate post-exercise recovery	Blunted redox adaptations	Allowed	Athletes engaged in high-intensity sports	[81,84]
		Curcumin	Group B	Moderate	A natural yellow polyphenol derived from turmeric rhizome	Attenuate post-exercise muscle soreness, reduce exercise-induce muscle damage, promote post-exercise recovery	Mild GI symptoms, nepatotoxicity	Allowed	Competitive athletes	[85,86]
		Probiotics	Group A	Moderate	Modulate gut barrier function and immune homeostasis	Reduce the risk of upper respiratory tract infections, alleviate exercise-induce gastrointestinal discomfort	Bloating, flatulence	Allowed	Competitive athletes	[88,89]
		Ashwagandha	-	Low	Adaptogenic herbs	Improve aerobic capacity, enhance muscular strength, promote post-exercise recovery	Drowsiness, hepatotoxicity	Allowed	Competitive athletes	[90]

Abbreviations: NaHCO_3_, sodium bicarbonate; EGCG, epigallocatechin-3-gallate; T_3_, triiodothyronine; AAS, anabolic–androgenic steroids; SARMs, selective androgen receptor modulators; 2,4-DNP, 2,4-dinitrophenol; AIS, Australian Institute of Sport; GRADE, Grading of Recommendations Assessment, Development and Evaluation; GI, gastrointestinal; HPG, hypothalamic–pituitary–gonadal.

## 3. Potential Risks of Sports Supplements

With the widespread use of sports supplements in competitive sports, their potential safety concerns have attracted increasing attention [92]. Existing studies have shown that sports supplements may exert adverse effects on multiple physiological systems [93]. Accordingly, the following section systematically summarizes their potential harms from six perspectives: disruption of endocrine homeostasis, adverse cardiovascular effects, gastrointestinal symptoms, hepatotoxicity, kidney injury, and supplement contamination and inadvertent anti-doping rule violations. In addition, given that evidence regarding the potential adverse effects of emerging sports supplements remains limited and their safety profiles have not yet been fully characterized, this review primarily focuses on the potential risks associated with long-term or high-dose use of classical sports supplements.

It is important to note that the substances discussed above under the broad umbrella of conventional sports supplements differ substantially in their regulatory status, legal implications, and level of scientific evidence. Accordingly, they can be broadly classified into four categories: evidence-based nutritional supplements, prescription drugs misused in sport, anabolic agents or agents with anabolic-like effects, and prohibited substances. Protein, creatine, carbohydrates, NaHCO_3_, vitamin D, vitamin C, iron, EGCG, and caffeine may be considered evidence-based nutritional supplements when used appropriately [47,94,95,96,97,98,99]. In contrast, T3 is a prescription drug that may be misused in sport, whereas AAS and SARMs are classified as anabolic agents or agents with anabolic-like effects [100,101,102]. Ephedrine and 2,4-DNP are classified as prohibited substances [103,104]. Clearly distinguishing among these categories provides an essential framework for understanding the potential risks associated with different sports-related substances in the following sections and helps prevent conceptual confusion among substances with fundamentally different regulatory and pharmacological profiles.

Moreover, despite the substantial differences in the regulatory and pharmacological profiles of these four categories of substances, their potential health risks should be systematically evaluated within an integrated analytical framework. There are several reasons for this approach. First, nutritional supplements, prescription drugs, anabolic-like compounds, and prohibited substances are all used by competitive athletes to enhance performance or modify body composition, with considerable overlap in their motivations for use, acquisition channels, and patterns of concurrent use [105]. Second, some commercially available sports supplements have been found to be adulterated with non-nutritional bioactive compounds, including AAS, SARMs, T_3_, and 2,4-DNP. Consequently, athletes may be unknowingly exposed to the combined risks of nutritional and non-nutritional substances, meaning that a narrow focus on compliant or legally marketed supplements would not adequately reflect real-world patterns of exposure [106]. Finally, incorporating these distinct categories into a unified analytical framework enables a more comprehensive characterization of their potential harms from public health and risk-assessment perspectives and provides an evidence base for tiered regulatory and risk-management strategies [107].

### 3.1. Endocrine Homeostasis Disruption

The endocrine system primarily relies on a precise feedback regulatory network composed of the hypothalamic–pituitary–target gland axes to maintain the dynamic balance of hormone levels in the body [108]. After absorption into the bloodstream, sports supplements may act on different levels of this regulatory network, thereby affecting the synthesis, secretion, and release of hormones, disrupting endocrine homeostasis, and further inducing reproductive dysfunction as well as structural and functional damage to related endocrine glands [109,110].

Long-term use of high-dose AAS is commonly associated with suppression of the hypothalamic–pituitary–gonadal (HPG) axis, which represents one of the most prevalent adverse effects reported in AAS users. Specifically, evidence from animal studies and athletic populations indicates that AAS increase circulating androgen and estradiol concentrations, thereby reducing the pulsatile secretion of gonadotropin-releasing hormone (GnRH) at the hypothalamic level, inhibiting the release of luteinizing hormone (LH) and follicle-stimulating hormone (FSH) from the anterior pituitary, and ultimately decreasing endogenous testosterone and estrogen production [109,110]. These alterations may lead to virilizing features, menstrual disturbances, and even amenorrhea in female athletes, while in male athletes they may result in testicular atrophy and impaired spermatogenesis [101,111]. Koltun et al. investigated women who exercised for 2 h per week and found that supraphysiological AAS use could induce menstrual disturbances and even amenorrhea. In that study, 17% of participants had low estrogen levels, which was presumed to be a consequence of AAS-induced suppression of the HPG axis [112]. It should be noted that this study employed a cross-sectional observational design, involved a relatively limited sample size, and included non-competitive participants who engaged in approximately 2 h of exercise per week. Therefore, caution is warranted when extrapolating these findings to competitive athletes who engage in long-term, high-dose AAS use [112]. In addition, an animal experimental study showed that after adult male rats were treated with AAS at doses of 10 mg/kg, 20 mg/kg, and 30 mg/kg for 8 weeks, testicular length, diameter, weight, and sperm count all decreased to varying degrees, and these adverse effects were positively associated with the administered AAS dose [113]. It should be noted that this study employed a Wistar rat model, which differs systematically from humans in androgen receptor distribution and reproductive physiology. Therefore, direct extrapolation of its dose–response findings to human athletes is subject to inherent limitations [113]. Furthermore, after discontinuation of AAS, recovery of HPG-axis function mainly depends on the duration of AAS use, dosage, and administration pattern [114]. Previous studies have shown that individuals with short-term or single-cycle AAS use may experience partial or complete recovery of HPG-axis function after cessation. However, long-term or repeated high-dose AAS use markedly increases the risk of delayed or incomplete recovery, and some individuals may develop irreversible hypogonadism [114,115].

Animal studies suggest that T_3_, as an exogenous thyroid hormone, can cross the blood–brain barrier and act on the hypothalamus and pituitary gland. Even when supplemented within an evidence-based therapeutic framework, it may disrupt endocrine homeostasis [100]. At the hypothalamic level, T_3_ binds to thyroid hormone receptors (TRs) and suppresses the production of thyrotropin-releasing hormone (TRH), thereby reducing pituitary thyroid-stimulating hormone (TSH) secretion. At the pituitary level, T_3_ can also directly inhibit TSH secretion [100,116]. The resulting decline in TSH levels further suppresses the release of thyroid hormones (THs), disrupting the negative-feedback homeostasis of the hypothalamic–pituitary–thyroid (HPT) axis and ultimately leading to hypothyroidism and glandular atrophy [116]. Furthermore, a case report showed that a 25-year-old male bodybuilder who ingested 25 μg of T_3_ daily for 4 consecutive weeks exhibited undetectable levels of TSH and free T_4_, accompanied by markedly elevated free T_3_ levels. These findings suggest that exogenous T_3_ intake can induce an abnormal thyroid function profile characterized by elevated T_3_, suppressed TSH, and reduced T_4_ levels. This case indicates that non-medical use of T_3_ may disrupt thyroid homeostasis by suppressing the HPT axis and reducing endogenous thyroid hormone production [117]. However, given the relatively small sample size, the generalizability of these findings to broader athletic populations remains to be further validated [117]. In addition, an analysis of 498 serum samples from elite Australian athletes suggested that supraphysiological T_3_ exposure may suppress TSH secretion via negative feedback within the HPT axis and contribute to biochemical thyrotoxicosis, raising concerns regarding its potential to perturb thyroid homeostasis [118].

Caffeine, as a nonselective adenosine receptor antagonist, may induce acute and transient activation of the hypothalamic–pituitary–adrenal (HPA) axis following a single high-dose intake; however, this effect has been observed only under high-dose consumption conditions. Animal studies and clinical trials have explored this mechanism, showing that caffeine blocks the binding of adenosine to hypothalamic adenosine A_1_ and A_2_A receptors, thereby relieving the inhibitory effect of adenosine on corticotropin-releasing hormone (CRH) neurons. This promotes pituitary secretion of adrenocorticotropic hormone (ACTH), which subsequently activates the MC2R–cAMP–PKA signaling pathway, leading to a transient increase in cortisol secretion in the adrenal cortex. The cortisol response typically peaks 60 min after caffeine ingestion and gradually returns toward baseline levels within the following several hours [94,119,120]. Notably, repeated caffeine exposure over the short term may delay cortisol release, indicating that its sustained regulatory effects are relatively limited. Conversely, chronic and frequent caffeine consumption may blunt HPA axis responsiveness through the development of caffeine tolerance, thereby diminishing caffeine-induced stimulation of cortisol synthesis and secretion. Collectively, these findings suggest that the long-term endocrine effects of caffeine may be modest [121]. Although cortisol plays a key regulatory role in mediating the body’s stress response, existing evidence indicates that excessive and persistent elevations in cortisol levels are closely associated with the onset and progression of psychiatric disorders such as anxiety and depression [122]. A randomized crossover trial in male soccer players showed that, compared with the placebo group, both the caffeinated coffee group and the caffeine powder group delivered in gelatin capsules exhibited significantly higher serum cortisol levels at 60 min after ingestion of caffeine at 3 mg/kg. No significant difference in cortisol levels was observed between the two caffeine intervention groups, suggesting that different delivery forms of caffeine may have comparable absorption rates and bioavailability in the body and therefore exert similar stimulatory effects on cortisol secretion. However, this study only assessed cortisol levels at a single time point (60 min after ingestion) and did not determine the duration of caffeine-induced cortisol elevation or its recovery kinetics [123]. In a randomized crossover study involving 24 professional rugby players, ingestion of 800 mg of caffeine 1 h before exercise increased resistance exercise-associated cortisol levels by approximately 52%, suggesting that high-dose caffeine may amplify the exercise-induced HPA axis stress response [124].

**Figure 1:**
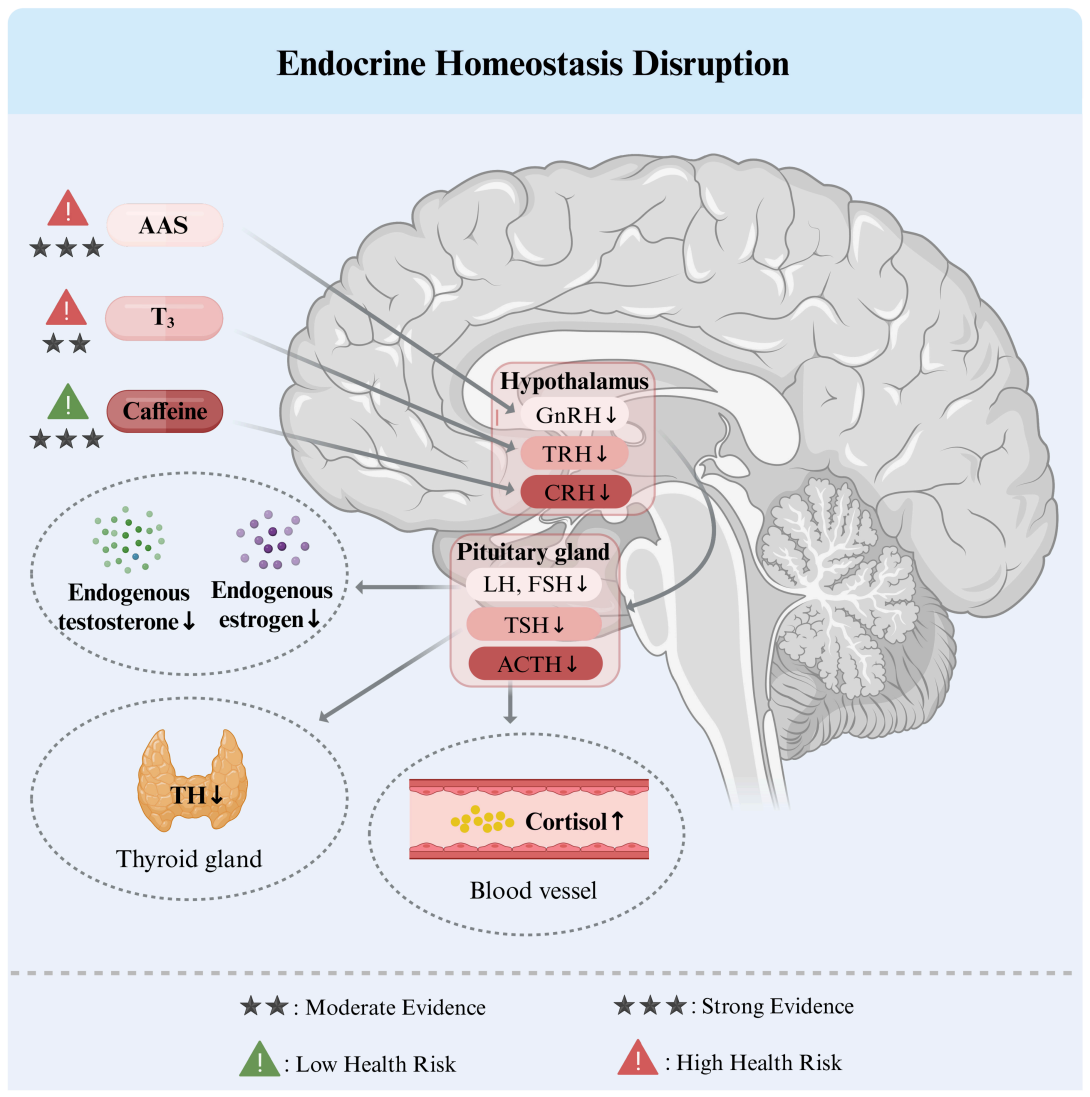
Endocrine homeostasis disruption induced by long-term and/or high-dose sports supplement use in athletes. Long-term and/or high-dose supplementation with sports supplements, including AAS, T_3_, and caffeine, in athletes may disrupt the normal physiological function of the hypothalamic–pituitary–target gland axes, thereby affecting normal hormone synthesis, secretion, and release and ultimately leading to endocrine homeostasis disruption. AAS, anabolic–androgenic steroids; T_3_, triiodothyronine; GnRH, gonadotropin-releasing hormone; TRH, thyrotropin-releasing hormone; CRH, corticotropin-releasing hormone; LH, luteinizing hormone; FSH, follicle-stimulating hormone; TSH, thyroid-stimulating hormone; ACTH, adrenocorticotropic hormone; TH, thyroid hormone; ↑, increase; ↓, decrease. Created with BioRender.com.

In summary, the adverse effects of long-term or high-dose intake of sports supplements on endocrine homeostasis are not limited to abnormal fluctuations in a single hormone level. Rather, they involve interference with the normal functions of multiple endocrine axes, including the HPG, HPT, and HPA axes, thereby disrupting hormone synthesis, secretion, and release at different regulatory levels and ultimately leading to systemic endocrine homeostatic disruption (Figure 1; Table 2).

### 3.2. Cardiovascular Adverse Effects

After being absorbed through the gastrointestinal tract into the bloodstream, sports supplements can be distributed via the systemic circulation to key cardiovascular structures, including myocardial tissue, coronary arteries, and peripheral blood vessels. They may further affect cardiovascular cholesterol levels, adrenergic receptor activity, and mitochondrial function in cardiomyocytes, thereby altering myocardial contractility, myocardial energy-metabolism homeostasis, and peripheral vascular tone, ultimately inducing a range of adverse cardiovascular effects [103,125].

Long-term or high-dose AAS supplementation can induce an atherogenic lipid profile and markedly increase the risk of atherosclerosis; however, this adverse effect has been primarily observed in individuals with long-term or high-dose AAS use. Its typical features include an approximately 20–70% reduction in high-density lipoprotein cholesterol (HDL-C), an approximately 20% increase in low-density lipoprotein cholesterol (LDL-C), and usually a moderate increase in total cholesterol [126,127]. This abnormal lipid profile is primarily driven by AAS-mediated disruption of hepatic cholesterol metabolism. Specifically, studies in athletic populations have shown that AAS can reduce the expression of proteins involved in HDL-C formation, such as apolipoprotein A-I, accelerate HDL-C catabolism, and promote LDL-C anabolism, thereby causing dyslipidemia and ultimately contributing to the development of atherosclerosis. This effect also shows a clear dose-dependent relationship with AAS exposure [127,128]. A study of 140 male weightlifters showed that long-term AAS use (continuous use for 2 years) may be associated with adverse coronary vascular remodeling. Compared with non-users, AAS users had a greater coronary artery plaque volume, and plaque volume was positively correlated with the duration of AAS use. This process was closely associated with AAS-induced abnormalities in the lipid profile [129]. After discontinuation of AAS, lipid levels may recover to some extent; however, withdrawal symptoms such as depression and hypogonadism may occur, and effective interventions are currently lacking. These factors may further increase the risk of cardiovascular disease [127]. Studies have shown that recreational athletes typically use AAS at supraphysiological doses, and that even a single cycle with a median duration of only 13 weeks is sufficient to increase LDL-C and decrease HDL-C, thereby producing a lipid profile with a pro-atherogenic pattern [130]. Additionally, a study of high-level bodybuilders reported similar findings: compared with bodybuilders who did not use AAS, those with long-term AAS use had significantly lower HDL-C levels and significantly higher LDL-C levels [131]. Collectively, these findings suggest that long-term or high-dose AAS use may promote the initiation and progression of atherosclerosis by inducing dysregulation of lipid metabolism.

In addition, the intake of sports supplements may also adversely affect blood pressure. Mostofsky et al. found that the relationship between caffeine intake and cardiovascular outcomes is generally nonlinear, and that the pressor effects of caffeine vary significantly depending on different intake patterns (short-term or long-term intake) [132]. For short-term caffeine intake, a single dose of approximately 200–300 mg caffeine can induce a transient increase in blood pressure within 1 h after ingestion, and this effect can persist for up to 3 h, with systolic and diastolic blood pressure increasing by approximately 8.1 mmHg and 5.7 mmHg, respectively [133]. In contrast, the hypertensive effects of long-term caffeine intake are influenced by multiple factors, including dosage, duration of intake, and the development of physiological tolerance. When daily caffeine intake exceeds 400 mg, the elevation in diastolic blood pressure becomes more pronounced; moreover, when the duration of intake exceeds 9 weeks, both systolic and diastolic blood pressure can be significantly increased [134]. Findings in athletic populations suggest that long-term continuous intake of caffeine at 400 mg/day can enhance sympathetic nervous system activity, promote the release of catecholamines such as norepinephrine, and activate α1-adrenergic receptors on vascular smooth muscle cells (VSMCs), thereby inducing vasoconstriction, increasing peripheral vascular resistance, and ultimately elevating blood pressure [134]. However, the magnitude of blood pressure elevation is also influenced by factors such as sex. Specifically, the pressor response is more pronounced in men than in women [134]. It is important to note that the hypertensive effect of caffeine described above has primarily been observed under conditions of long-term or high-dose intake and may represent a toxicological response rather than an effect associated with typical caffeine consumption.

Studies in athletic populations have shown that ephedrine, as a sympathomimetic amines, can enhance sympathetic nervous system excitability and promote the release of norepinephrine from sympathetic nerve terminals, thereby activating α_1_-, β_1_-, and β_2_-adrenergic receptors. This process can increase heart rate and myocardial contractility, while α_1_-receptor-mediated peripheral vasoconstriction increases peripheral vascular resistance, aggravates left ventricular afterload, and raises myocardial oxygen demand, thereby increasing the risk of myocardial ischemia. In addition, norepinephrine may induce coronary artery spasm, reduce myocardial blood perfusion, and further exacerbate ischemic myocardial injury, which in severe cases may progress to myocardial infarction [104]. This adverse effect has primarily been reported in athletes who misuse ephedrine. Multiple case reports have shown that athletes may develop varying degrees of myocardial ischemia or even myocardial infarction after ephedrine use. Under stress conditions such as dehydration, elevated body temperature, and exercise, this type of myocardial injury may be further aggravated. These case reports also indicate that there is no clear linear relationship between the dose of ephedrine and adverse cardiovascular reactions; even at low doses, susceptible individuals may experience myocardial injury [135,136,137]. In addition, a case report by Naik et al. described ephedrine-associated cardiovascular injury, showing that ephedrine ingestion was followed by impaired left ventricular systolic function, with the ejection fraction declining to 40–50%. The authors further noted that, through its sympathomimetic effects, ephedrine increases myocardial oxygen demand and imposes greater stress on the coronary circulation, thereby predisposing individuals to a range of adverse cardiovascular events, including myocardial ischemia and even myocardial infarction [138]. It should be noted that this conclusion is based on a single case report and therefore provides limited support for causal inference. In addition, concomitant use of other stimulants, the patient’s baseline cardiovascular status, and the actual dose of ephedrine exposure could not be accurately determined. Accordingly, caution is warranted when extrapolating these findings directly to athletic populations [138]. Therefore, because of its severe cardiovascular toxicity, ephedrine was banned for sale by the U.S. Food and Drug Administration (FDA) in 2004 [104].

2,4-DNP is a synthetic mitochondrial uncoupler that has been misused by bodybuilders for fat loss. Experimental studies have shown that it primarily enhances mitochondrial uncoupling in cardiomyocytes, causing the proton gradient generated by the electron transport chain across the inner mitochondrial membrane to be dissipated as heat rather than being used to drive adenosine 5′-triphosphate (ATP) synthesis. This induces a hypermetabolic and hyperthermic state. Because its effective dose is extremely close to its lethal dose, these toxic reactions can be readily triggered, leading to persistent tachycardia, acute heart failure, and even sudden death [103]. It should be noted that the toxic effects associated with 2,4-DNP have primarily been reported in athletes who misuse this compound. A case report of a young bodybuilder with muscle dysmorphia showed that, after repeatedly taking 2,4-DNP for approximately 6 months, he died following another intake of 2 g of the substance. Six months before his death, he had already developed typical hypermetabolic and hyperthermia-related symptoms, including tachypnea, profuse sweating, and tachycardia, and 4 months before death, he progressed to multiple organ failure [139]. This suggests that the toxic effects of 2,4-DNP are not limited to the heart but may further accumulate across multiple organ systems. Additionally, a case report of a novice bodybuilder described fatal intoxication following the ingestion of 2,4-DNP and clenbuterol. Subsequent toxicological analysis revealed high concentrations of both 2,4-DNP and clenbuterol in his blood and urine. Based on the circumstances in which the body was found and the toxicological findings, the authors attributed the cause of death to fatal hyperthermia induced by 2,4-DNP ingestion [140]. It should be noted that the evidence described above was derived exclusively from uncontrolled reports of fatal cases. Moreover, the athletes were concurrently exposed to clenbuterol and multiple anabolic agents, making it difficult to determine the toxicity specifically attributable to 2,4-DNP or to establish a reliable dose–response relationship. In addition, these cases all involved a relatively atypical subset of bodybuilders; therefore, these findings cannot be directly generalized to the broader athlete population. Larger-scale studies are needed to determine the incidence and severity of the associated risks [139,140].

**Figure 2:**
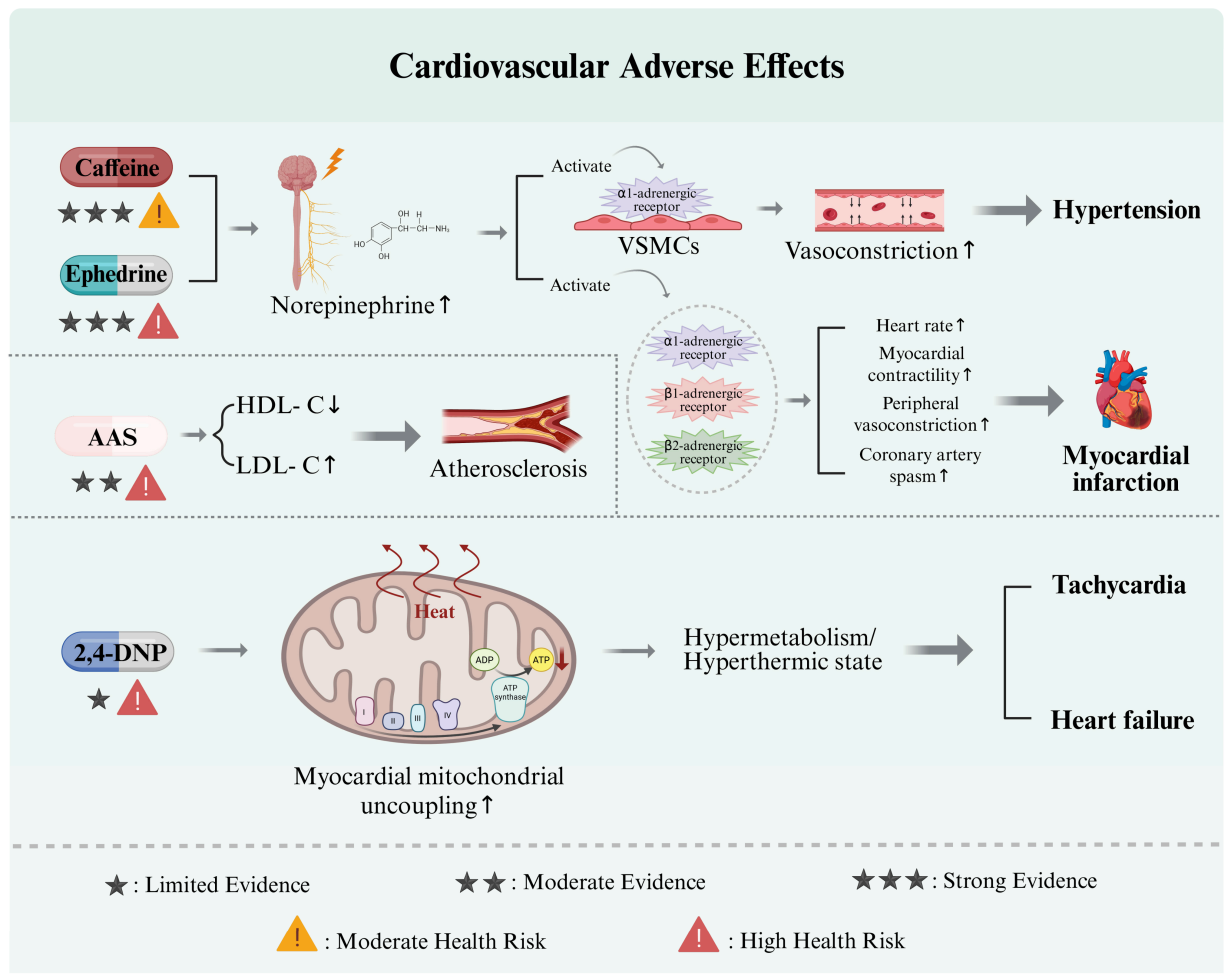
Cardiovascular adverse effects induced by long-term and/or high-dose sports supplement use in athletes. Long-term and/or high-dose supplementation with sports supplements, including caffeine, ephedrine, AAS, and 2,4-DNP, in athletes may induce cardiovascular adverse effects, such as hypertension, myocardial infarction, tachycardia, and heart failure, by enhancing sympathetic nervous system excitability, inducing an atherogenic lipid profile, and promoting mitochondrial uncoupling in cardiomyocytes. VSMCs, vascular smooth muscle cells; AAS, anabolic–androgenic steroids; HDL-C, high-density lipoprotein cholesterol; LDL-C, low-density lipoprotein cholesterol; 2,4-DNP, 2,4-Dinitrophenol; I, NADH: ubiquinone oxidoreductase; II, succinate: ubiquinone oxidoreductase; III, ubiquinol: cytochrome c oxidoreductase; IV, cytochrome c oxidase; ADP, adenosine diphosphate; ATP, adenosine triphosphate; ↑, increase; ↓, decrease. Created with BioRender.com.

In summary, long-term or high-dose intake of sports supplements may induce a range of cardiovascular diseases, including coronary atherosclerosis, hypertension, myocardial ischemia, myocardial infarction, and tachycardia, through mechanisms such as the induction of an atherogenic lipid profile, enhancement of sympathetic nervous system excitability, and mitochondrial uncoupling in cardiomyocytes (Figure 2; Table 2).

### 3.3. Gastrointestinal Symptoms

As the primary site of contact after oral intake of sports supplements, the gastrointestinal tract allows direct interaction between the mucosal epithelium and the active components of these supplements. This interaction may affect gastrointestinal mucus-layer homeostasis, regulate sympathetic nervous system excitability, alter intestinal osmotic pressure, and modify the composition of the gut microbiota, ultimately impairing the normal structure and function of the gastrointestinal tract [95,96,141,142].

Acute caffeine ingestion at evidence-based doses in athletes may adversely affect gastrointestinal barrier function, representing a commonly reported adverse effect of caffeine supplementation. Experimental studies suggest several potential mechanisms, as outlined below. Caffeine may inhibit gastric mucin secretion, thereby disrupting gastric mucus-layer homeostasis and weakening the protective function of the gastric mucus barrier [142]. At the same time, caffeine may stimulate colonic motility and promote intestinal fluid secretion, increasing mechanical load within the intestinal lumen and damaging the intestinal mucus layer [143]. In addition, as an adenosine receptor antagonist, caffeine can relieve the inhibitory effect of adenosine on sympathetic activity, further enhancing catecholamine release under exercise-induced stress. This may aggravate visceral vasoconstriction, promote the redistribution of blood flow from the splanchnic circulation to skeletal muscle during exercise, and ultimately lead to insufficient gastrointestinal perfusion and intestinal barrier dysfunction [144]. These mechanisms may occur independently or synergistically, and their effects vary substantially among individuals due to factors such as genetic differences [145]. A randomized crossover trial involving 18 young healthy cyclists demonstrated that, under standardized conditions of overnight fasting, abstinence from caffeine intake for 48 h, and avoidance of strenuous exercise for 24 h before the experiment, participants ingested either caffeine (3 mg/kg body mass) or placebo 45 min before cycling exercise. They subsequently performed a 20-min cycling bout at an intensity corresponding to 70% of maximal oxygen uptake, followed by a 15-min cycling time trial after a brief recovery period. Compared with placebo, caffeine ingestion resulted in a significant increase in post-exercise plasma levels of intestinal fatty acid-binding protein (iFABP), a biomarker of intestinal epithelial cell injury, suggesting that acute caffeine intake may exacerbate endurance exercise-induced intestinal epithelial damage and impair intestinal barrier integrity [145].

In addition, carbohydrate intake at evidence-based doses in athletes may also induce gastrointestinal symptoms, and its occurrence is related to factors such as carbohydrate type and solution osmolality; this represents a common adverse effect of carbohydrate supplementation [95]. Evidence from studies in athletes indicates that hypertonic carbohydrate solutions can create an osmotic gradient within the intestinal lumen, promoting the movement of water from plasma and interstitial fluid into the intestinal lumen. This leads to intestinal distension and ultimately triggers gastrointestinal symptoms such as bloating and diarrhea [95]. A survey study of 55 male triathletes revealed an association between ingestion of hypertonic carbohydrate solutions and gastrointestinal symptoms. Among athletes who consumed hypertonic beverages, 40% reported severe gastrointestinal symptoms, including flatulence, abdominal bloating, diarrhea, nausea, vomiting, and intestinal cramps, a rate substantially higher than that observed among those consuming isotonic or hypotonic beverages. These findings suggest that pre-race ingestion of hypertonic solutions may provoke or exacerbate exercise-induced gastrointestinal distress by delaying gastric emptying and increasing the osmotic load within the intestinal lumen [146]. In addition, evidence from both field and laboratory studies consistently indicates that ingestion of highly hypertonic beverages before exercise may elicit a range of gastrointestinal symptoms, including stomach pain, vomiting, reflux, and heartburn. In contrast, there is currently no evidence that consuming non-hypertonic beverages during exercise induces gastrointestinal discomfort [147].

NaHCO_3_ is one of the sports supplements with relatively poor gastrointestinal tolerability. The ISSN suggests that supplementation with 0.3 g/kg NaHCO_3_ can provide the greatest ergogenic benefit, whereas higher doses, such as 0.4 g/kg or 0.5 g/kg, are more likely to cause side effects including bloating, nausea, vomiting, and abdominal pain. Experimental studies and findings in athletic populations have provided mechanistic insight into these effects. Specifically, NaHCO_3_ is highly soluble in gastric fluid, and high-dose NaHCO_3_ can rapidly dissociate in the stomach into large amounts of Na^+^ and HCO_3_^−^. HCO_3_^−^ then reacts with hydrochloric acid (HCl) to generate a large amount of carbon dioxide (CO_2_), causing intragastric gas retention and increasing gastric distension, thereby inducing bloating, upper abdominal discomfort, and even nausea and vomiting [47]. At the same time, because the amount of NaHCO_3_ exceeds the amount of gastric HCl, the ingested high dose of NaHCO_3_ does not completely react with gastric acid [47]. The unreacted Na^+^ and HCO_3_^−^ can create a hypertonic environment within the intestinal lumen, driving water movement into the intestinal cavity along an osmotic gradient. This increases intestinal contents and causes luminal distension, leading to osmotic diarrhea and abdominal cramping [47]. The incidence and severity of these adverse reactions show a clear dose-dependent pattern [148]. A randomized, double-blind, crossover trial involving 25 male elite rugby players showed that NaHCO_3_ supplementation, even at the ISSN-recommended dose of 0.3 g/kg, has a dual effect. On the one hand, NaHCO_3_ significantly increased blood HCO_3_^−^ concentrations and effectively attenuated the decline in blood pH during high-intensity exercise. On the other hand, this metabolic alkalinization did not translate into a significant improvement in exercise performance and was instead accompanied by gastrointestinal symptoms, including stomach pain, bloating, nausea, and vomiting, with a significantly higher incidence than in the placebo condition [149]. Durkalec-Michalski et al. demonstrated in a randomized, double-blind crossover trial that NaHCO_3_ supplementation elicited dose-dependent gastrointestinal discomfort when doses were scaled to fat-free mass (FFM) (0.15, 0.25, and 0.35 g·kg^−1^ FFM). Notably, the highest dose produced significantly greater gastrointestinal discomfort than the control, placebo, and low-dose conditions, with similar responses observed in both male and female athletes [150].

The gastrointestinal tract is the primary site of microbial colonization in the body and contains more than approximately 70% of the human microbial community [151]. Animal studies, human trials, and in vitro experiments have shown that high-dose oral iron supplementation can directly disrupt the composition of the gut microbiota, characterized by reduced abundances of beneficial bacteria such as *Lactobacillus*, *Bifidobacterium*, and *Faecalibacterium*, together with increased abundances of pathogenic bacteria such as *Salmonella*, *Shigella*, and *Campylobacter*. In addition, excess iron can enhance the virulence of these pathogenic bacteria, ultimately increasing the risk of intestinal inflammation [96]. Furthermore, excess iron can promote the generation of ROS within the intestinal lumen and induce oxidative stress, thereby damaging the integrity of the intestinal epithelial barrier and further aggravating intestinal inflammation [96]. It is important to note that intestinal inflammatory responses induced by iron supplementation have only been reported under conditions of high-dose iron intake. A study investigating the effects of different iron statuses on intestinal inflammation in mice found that, compared with the iron-deficient and iron-normal groups, mice in the iron-overload group exhibited significant colonic inflammation. Further analysis indicated that this phenomenon was closely associated with iron-overload-induced gut microbiota dysbiosis and enhanced oxidative stress responses [152]. It should be noted that this study was conducted in a mouse model, and species-specific differences in iron metabolism and gut microbiota composition limit the direct extrapolation of its mechanistic findings and estimated risk magnitude to human athletes. These findings therefore require validation in clinical studies involving athletic populations [152]. In addition, McCormick et al. noted in their review that oral iron, although considered a first-line supplementation strategy for iron-deficient athletes, is often limited by gastrointestinal side effects at higher doses. In athletes with gastrointestinal sensitivity, high-dose iron supplementation can readily cause gastric irritation and may even contribute to intestinal inflammation, thereby compromising treatment efficacy [153].

**Figure 3:**
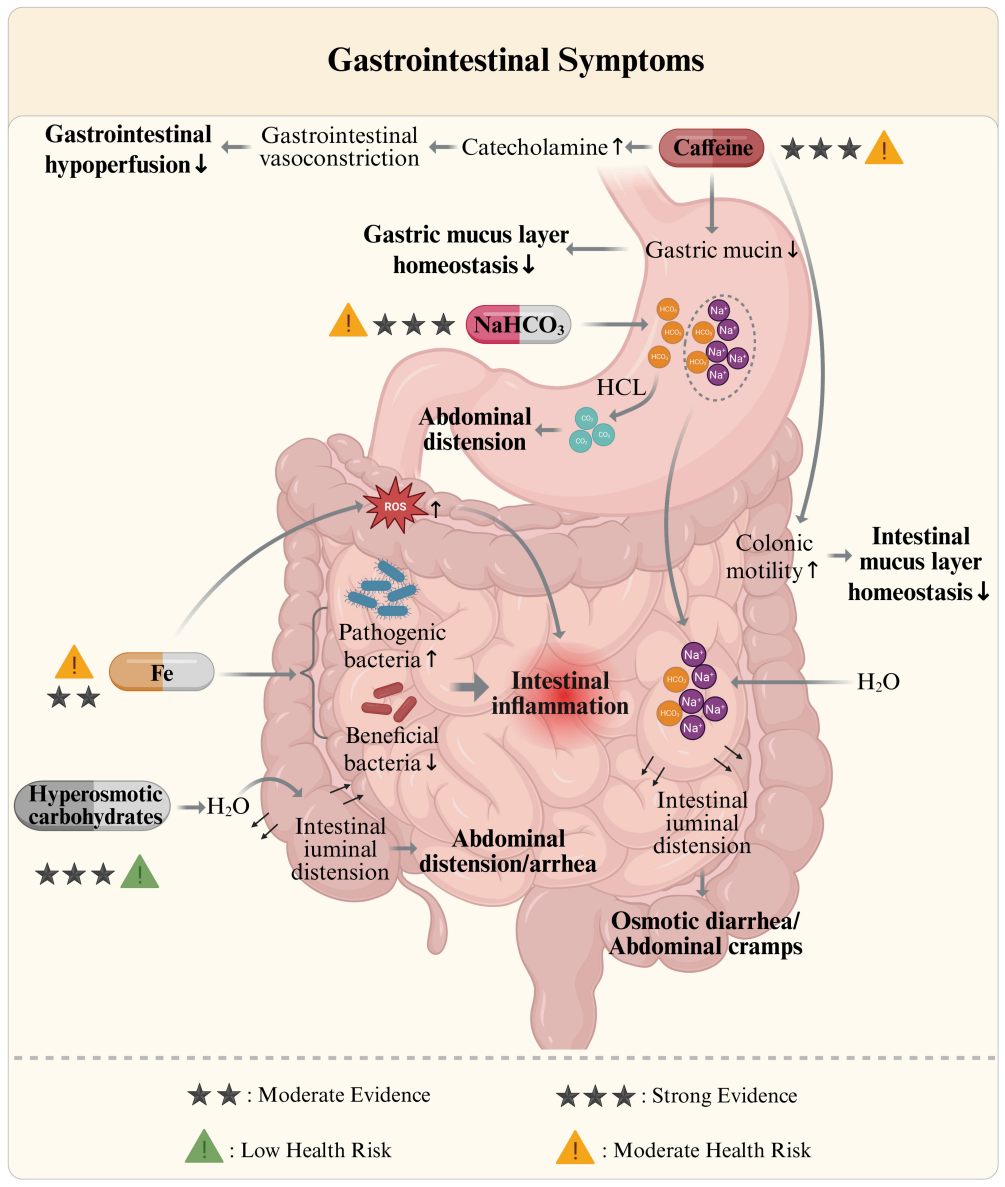
Gastrointestinal symptoms induced by long-term and/or high-dose sports supplement use in athletes. Long-term and/or high-dose supplementation with sports supplements, including caffeine, NaHCO_3_, Fe, and hypertonic carbohydrates, in athletes may induce gastrointestinal symptoms, such as bloating, diarrhea, abdominal cramps, and intestinal inflammation, by impairing gastrointestinal barrier function, promoting intestinal luminal distension, disrupting gut microbiota composition, and increasing ROS generation in the intestinal lumen. NaHCO_3_, sodium bicarbonate; Na^+^, sodium ion; HCO_3_^−^, bicarbonate ion; HCL, hydrochloric acid; CO_2_, carbon dioxide; Fe, iron; ROS, reactive oxygen species; ↑, increase; ↓, decrease. Created with BioRender.com.

Thus, high-dose sports supplements may ultimately lead to gastrointestinal symptoms such as gastrointestinal barrier dysfunction, bloating, diarrhea, abdominal pain, and intestinal inflammation through mechanisms including enhanced sympathetic nervous system excitability, increased intestinal osmolality, intragastric gas retention, disruption of gut microbiota composition, and induction of oxidative stress (Figure 3; Table 2).

### 3.4. Hepatotoxicity

The liver is the central organ responsible for the metabolism of sports supplements. After absorption, most orally ingested sports supplements enter the liver through the portal vein and undergo biotransformation within hepatocytes [154]. Continuous exposure of hepatocytes to sports supplements may affect the rate of ROS generation, mitochondrial health, bile secretion and transport, and serum transaminase levels, thereby impairing the normal structure and function of the liver [155].

Animal and in vitro studies have shown that the hepatotoxicity of AAS and EGCG is mainly driven by hepatic oxidative stress [156,157]. During supplementation, the rate of ROS generation in hepatocytes rises sharply and is positively associated with the supplementation dose. Therefore, increased ROS production during sports supplement use can directly lead to liver injury, manifested as hepatocyte apoptosis or necrosis [158]. For example, studies have found that athletes and bodybuilders may abuse AAS to increase muscle mass. These synthetic testosterone analogs can bind to and activate androgen receptors (ARs), which are widely expressed in hepatocytes. On the one hand, AR activation promotes excessive ROS production; on the other hand, it upregulates mitochondrial β-oxidation. Together, these processes exacerbate oxidative stress-mediated hepatocellular injury, providing a plausible mechanistic explanation for sports supplement-induced hepatotoxicity [156]. Pahlavani et al. reached a similar conclusion, noting that the liver is one of the primary target organs of AAS-induced toxicity. In some young athletes, AAS abuse may disrupt hepatic redox homeostasis and promote oxidative stress-mediated hepatocellular injury [159]. An animal study based on EGCG supplementation in mice showed that a single administration of 1500 mg/kg EGCG increased plasma alanine aminotransferase (ALT) levels by 138-fold. Under conditions of once-daily administration of 750 mg/kg EGCG, plasma ALT levels increased by 184-fold after two doses. These findings suggest that EGCG intake dose may be a key factor influencing hepatotoxicity, while its liver-injury effects may also show a degree of time dependence and cumulative effect. This change was further presumed to be related to hepatic oxidative stress [160]. It should be noted that these findings were derived from acute or subacute high-dose gavage studies in mice. Given the marked interspecies differences between mice and humans in EGCG metabolism, bioavailability, and detoxification capacity, as well as the substantially higher doses used relative to typical human intake, caution is warranted when extrapolating these findings to human athletes. The hepatotoxic risk of EGCG in athletes therefore requires further validation through clinical studies [160].

However, animal studies indicate that high-dose EGCG-induced hepatotoxicity is not limited to oxidative stress mechanisms; it also involves oxidative stress-independent mechanisms of mitochondrial injury in hepatocytes. This may be related to disruption of the mitochondrial membrane potential, which can increase mitochondrial membrane permeability and thereby damage hepatocytes [161]. Animal studies have shown that EGCG, at concentrations ranging from 10 to 500 μM, was used to assess its short-term effects on hepatic mitochondrial function and induced mild uncoupling of hepatic mitochondria, thereby disrupting the mitochondrial membrane potential and promoting oxidation of reduced nicotinamide adenine dinucleotide (NADH) by the electron transport chain. This shifts the NADH/NAD^+^ ratio toward a more oxidized state, and the resulting change in redox status increases mitochondrial membrane permeability, ultimately leading to hepatocyte injury [97]. A positive feedback loop may therefore form between oxidative stress and mitochondrial injury: oxidative stress directly damages mitochondria, while damaged mitochondria further release ROS due to electron transport chain dysfunction. These two processes act synergistically to aggravate liver injury and generate a cascade-amplification effect. In addition, an in vitro study evaluated the hepatotoxic effects of EGCG in rat hepatocytes. Using primary cultured rat hepatocytes and digitonin-permeabilized hepatocytes as experimental models, the study exposed cells to 5–100 μmol/L EGCG for 24 h. The results showed that EGCG impaired hepatocellular function even at 10 μmol/L, in a dose-dependent manner. At higher concentrations, EGCG further reduced mitochondrial membrane potential and exacerbated hepatocellular injury [162]. It should be noted that all of the studies described above were conducted in animal models. Given the substantial interspecies differences between humans and animals in EGCG metabolism, bioavailability, and detoxification capacity, caution is warranted when extrapolating these findings directly to human athletes [97,162].

For SARMs, hepatotoxicity may be less influenced by hepatocellular oxidative stress and mitochondrial injury and may instead be more closely related to impaired bile excretion [102]. Based on previous animal studies of the mechanisms underlying AAS-induced cholestasis, it has been proposed that SARMs may impair bile excretion through two pathways. On the one hand, they inhibit bile acid efflux mediated by the bile salt export pump (BSEP), thereby weakening bile salt-dependent bile flow. On the other hand, they reduce hepatocyte membrane permeability to osmotically driven factors, suppressing bile salt-independent bile flow. These changes are accompanied by structural damage to bile canalicular microvilli and canalicular lumen dilation, ultimately inducing cholestatic liver injury [102]. A large body of evidence has confirmed that high-dose or long-term intake of SARMs is more likely to impair bile excretion and cause hepatic cholestasis [155,163]. Mohideen et al. summarized 12 cases of SARM-associated drug-induced liver injury (DILI). The patients were predominantly male fitness enthusiasts aged 19–52 years, who typically developed cholestatic manifestations, including jaundice and hyperbilirubinemia, approximately 4–12 weeks after continuous SARM use. Liver function gradually recovered after SARM discontinuation, suggesting that SARMs can induce liver injury characterized primarily by cholestasis [102]. Subsequently, Leciejewska et al. conducted a systematic review of 20 cases of SARM-related liver injury. These events occurred primarily in young male fitness enthusiasts aged 18-52 years. The review showed that these individuals commonly developed cholestatic liver injury after 14 days to 24 weeks of SARM use, and that liver function gradually returned to normal after SARM discontinuation [163]. However, the reported cases were largely confined to fitness-oriented individuals using SARMs for muscle gain, and the applicability of these findings to broader athletic populations remains to be further established [102,163].

In addition, creatine, as one of the most widely used sports supplements, is generally considered to have good hepatic safety when used at conventional doses [164]. However, animal studies have shown that high-dose or long-term creatine intake may induce hepatotoxicity, which may be associated with elevated serum transaminase levels. Increased serum transaminase levels usually indicate hepatocellular injury; such injury may be reversible in the short term but can progress to irreversible liver damage, such as liver cirrhosis, over the long term [165,166]. Animal studies have indicated that prolonged exposure to high-dose creatine may alter liver-related biochemical parameters. Specifically, Wistar rats receiving creatine at 1 g/kg/day for 4–8 weeks showed significantly increased serum transaminase levels [167]. However, because this study was conducted in Wistar rats, potential interspecies differences in creatine metabolism and exposure levels warrant caution when extrapolating these findings to human athletes [167]. Consistent with these findings, clinical studies have also suggested that long-term ingestion of high-dose creatine may lead to elevated serum transaminase levels, indicating that its potential hepatic safety risks may be dose- and time-dependent [168]. Given that the study participants had pre-existing medical conditions, the generalizability of these findings to broader athletic populations remains limited [168]. In addition, some studies have found that when creatine is used in combination with ethanol, it may impair liver structure and function by upregulating the expression of genes related to oxidative stress and inflammatory responses, as well as increasing serum transaminase levels [165].

**Figure 4:**
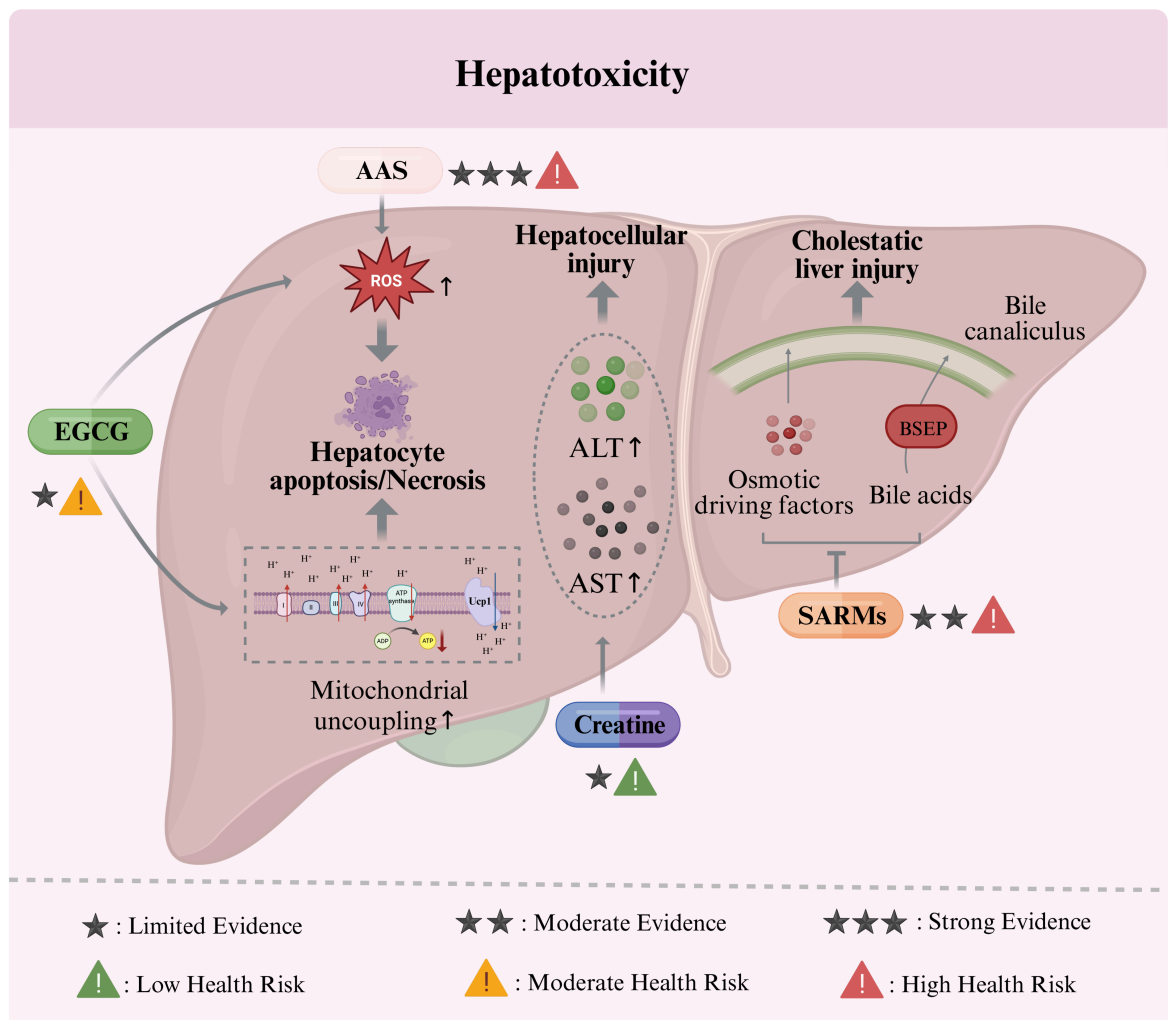
Hepatotoxicity induced by long-term and/or high-dose sports supplement use in athletes. Long-term and/or high-dose supplementation with sports supplements, including AAS, EGCG, creatine, and SARMs, in athletes may induce hepatotoxicity, such as hepatocyte apoptosis, necrosis, and cholestatic liver injury, by promoting ROS generation and mitochondrial uncoupling in hepatocytes, increasing serum transaminase levels, and impairing bile excretion. AAS, anabolic–androgenic steroids; EGCG, epigallocatechin gallate; ROS, reactive oxygen species; I, NADH: ubiquinone oxidoreductase; II, succinate: ubiquinone oxidoreductase; III, ubiquinol: cytochrome c oxidoreductase; IV, cytochrome c oxidase; ADP, adenosine diphosphate; ATP, adenosine triphosphate; Ucp1, uncoupling protein 1; H^+^, hydrogen ion; ALT, alanine aminotransferase; AST, aspartate aminotransferase; SARMs, selective androgen receptor modulators; BSEP, bile salt export pump; ↑, increase; ↓, decrease. Created with BioRender.com.

In summary, long-term or high-dose intake of sports supplements can impair liver structure and function through multiple pathological mechanisms, including aggravation of oxidative stress, increased mitochondrial membrane permeability, impaired bile excretion, and upregulation of serum transaminase levels. Moreover, some of these mechanisms may overlap and form positive feedback effects, indicating that supplement-induced hepatotoxicity is a complex pathological process driven by the coordinated action of multiple mechanisms and targets (Figure 4; Table 2).

### 3.5. Kidney Injury

Most sports supplements are eliminated mainly through urine and bile, with the kidneys serving as the primary organs mediating their excretion [169]. After sports supplements and their metabolites reach the kidneys via the systemic circulation, they are cleared sequentially through three processes: glomerular filtration, tubular secretion, and tubular reabsorption [170]. However, during this process, sports supplements and their metabolites may affect renal blood flow, glomerular filtration rate, and oxidative stress, thereby damaging the normal structure and function of the kidneys [21,171,172].

Vitamin supplements are among the most widely used sports supplements in athletes and are also one of the most extensively studied supplement categories, with representative examples including vitamin D and vitamin C [98]. The International Olympic Committee (IOC) explicitly stated in its consensus statement that high-dose vitamin D supplementation in athletes is closely associated with the occurrence of kidney stones [22]. Human studies have shown that vitamin D is converted into its active form, 1,25-dihydroxyvitamin D (calcitriol), through two sequential steps: 25-hydroxylation in the liver and 1α-hydroxylation in the kidney. Calcitriol can markedly enhance intestinal calcium absorption, leading to increased serum calcium levels [173]. Because the amount of calcium absorbed from the gastrointestinal tract into the bloodstream is positively correlated with the amount of calcium excreted by the kidneys, elevated serum calcium levels can directly increase urinary calcium excretion and induce hypercalciuria [173]. Hypercalciuria is one of the major determinants of calcium-dependent kidney stone formation. Excessive Ca^2+^ in the urine can promote abnormal deposition of calcium salts, such as calcium phosphate and calcium oxalate, within the kidney, ultimately increasing the risk of kidney stone formation [173]. Letavernier et al. reported that long-term or high-dose vitamin D supplementation in athletes may increase urinary calcium excretion by enhancing intestinal calcium absorption, thereby inducing or exacerbating hypercalciuria in susceptible individuals and further promoting kidney stone formation. When combined with calcium supplements, this risk appears to increase synergistically [173]. A case report showed that daily combined supplementation with calcium and vitamin D for two consecutive years, together with high-dose cholecalciferol at 60,000 IU once or twice monthly, induced vitamin D toxicity. This subsequently led to hypercalcemia and hypercalciuria, ultimately contributing to the formation of bilateral calcium oxalate kidney stones [174]. It should be noted that the patient described in this case report had diabetes mellitus, which may have affected vitamin D metabolism differently compared with that of athletes. Therefore, further validation is required before extrapolating these findings to athletic populations [174].

High-dose vitamin C may also induce kidney injury through mechanisms partly similar to those of vitamin D. Vitamin C is mainly metabolized in the liver, first undergoing nonenzymatic reversible oxidation to dehydroascorbic acid (DHA), and then irreversible hydrolysis mediated by 2,3-diketogulonic acid decarboxylase to form 2,3-diketogulonic acid (2,3-DKG). The latter is subsequently converted into oxalate through multiple oxidation reactions in the L-xylulose pathway [175]. Human pharmacokinetic studies and clinical case reports have shown that long-term high-dose vitamin C supplementation, with an adult daily upper intake limit of 1–2 g, can cause abnormal elevation of oxalate concentrations in the body, exceeding the kidney’s normal clearance capacity. As a result, large amounts of oxalate in the renal tubular lumen bind to Ca^2+^ to form calcium oxalate crystals, which deposit in the renal tubules and interstitium, thereby inducing oxalate nephropathy [175,176,177]. A recent case report analysis showed that continuous daily intake of 3 g vitamin C for 6 months increased calcium oxalate crystal deposition in renal tissue, a phenomenon considered likely related to high-dose vitamin C-induced hyperoxaluria [175]. Gurm et al. reported that daily vitamin C intake of approximately 3–6.5 g for 1 month was sufficient to generate oxalate through metabolism and induce intratubular calcium oxalate crystal deposition, accompanied by tubular atrophy and interstitial fibrosis, ultimately progressing to end-stage renal disease. The study further noted that renal injury remained irreversible even after vitamin C discontinuation, leaving the patient dependent on long-term hemodialysis [178]. It should be noted that the available evidence is derived solely from individual case reports, and all reported subjects had pre-existing medical conditions. These underlying health conditions may result in differences in vitamin C metabolism and physiological status compared with athletes; therefore, further validation is required before extrapolating the potential risk of renal injury to athletic populations [175,178].

Protein supplementation is extremely common among bodybuilders. Among them, the average daily protein supplementation can reach approximately 4.3 g/kg in male bodybuilders and approximately 2.8 g/kg in female bodybuilders, substantially exceeding the ISSN-recommended intake range of 1.4–2.0 g/kg/day [99,179]. However, long-term maintenance of such high-dose protein supplementation may impair kidney function to some extent. Specifically, animal studies and human trials have shown that, after high protein intake, the reabsorption of large amounts of amino acids in the proximal tubule is accompanied by increased Na^+^ cotransport, which lowers the sodium chloride (NaCl) concentration at the macula densa. This subsequently mediates afferent arteriolar dilation through the tubuloglomerular feedback (TGF) mechanism. At the same time, protein intake can stimulate glucagon secretion, directly promoting renal vasodilation [171]. Together, these two processes lead to compensatory increases in renal blood flow and glomerular filtration rate (GFR), facilitating the excretion of nitrogenous waste products [171]. However, sustained hyperfiltration and increased intraglomerular hydrostatic pressure can impose abnormal mechanical stress on capillary endothelial cells and podocytes, causing structural damage to the glomerulus. Meanwhile, the high-load excretion of nitrogenous waste further increases the metabolic burden on renal tubules [180,181,182]. Cho et al. reported that long-term high-protein intake in athletes may increase renal blood flow through vasodilatory mechanisms and induce glomerular hyperfiltration. At the same time, it can activate multiple endocrine and paracrine mediators, such as glucagon, insulin-like growth factor 1 (IGF-1), and nitric oxide, as well as pro-inflammatory cytokines, thereby promoting progressive glomerulosclerosis and impairing normal renal physiological function [171]. An animal study showed that, after rats were fed low-protein (4.6% casein), standard-protein (23% casein), or high-protein (57.5% casein) diets, those in the high-protein group developed marked proteinuria, azotemia, elevated serum creatinine, and reduced creatinine clearance, together with histological evidence of renal injury, including segmental glomerular proliferation and sclerosis. In contrast, these functional and histological abnormalities were not observed in the low-protein group. These findings suggest that long-term high-protein intake may impair renal function by increasing the glomerular workload, whereas moderate protein restriction may confer a degree of renal protection [183]. It should be noted that this study was conducted in a rat model, and protein metabolism may differ between rats and athletes. Therefore, the validity of extrapolating these findings to athletic populations requires further confirmation [183].

In addition to the above sports supplements, ephedrine may also damage the kidneys of athletes. Studies have shown that ephedrine supplementation, even at evidence-based doses, can induce rhabdomyolysis by enhancing sympathetic nervous system excitability [184]. This indicates that renal injury represents a common adverse effect associated with ephedrine use. Animal studies have shown that damaged skeletal muscle releases large amounts of myoglobin into the bloodstream, which then enters the renal tubules after glomerular filtration [185]. Within the renal tubular lumen, the heme contained in myoglobin releases free Fe^2+^, which generates ROS through the Fenton reaction and causes oxidative injury to renal tubular epithelial cells. At the same time, myoglobin can bind to Tamm–Horsfall protein (THP) to form cast precipitates, obstructing the distal tubular lumen [185]. These two mechanisms—oxidative injury and tubular obstruction—act synergistically and can lead to acute kidney injury (AKI), and in severe cases, tubular necrosis [185]. A case report involving an amateur marathon runner showed that the athlete collapsed during a race after taking 60 mg of ephedrine before the event and subsequently developed acute renal failure due to rhabdomyolysis [135]. The report further noted that exercise-induced rhabdomyolysis usually occurs in healthy individuals performing strenuous exercise under dehydrated conditions [135]. A case report described a previously healthy woman who developed severe thigh pain and cola-colored urine 48 h after a routine spinning session performed after taking an ephedra-containing herbal product, whose active ingredient is ephedrine. Laboratory testing showed marked elevations in creatine kinase and myoglobin, consistent with a diagnosis of rhabdomyolysis. This case suggests that, in exercising individuals, the use of ephedrine-containing supplements or herbal products during periods of high-intensity exercise may precipitate rhabdomyolysis and thereby cause secondary renal injury [186]. However, whether the effects observed in these case reports also occur among competitive athletes remains to be established through further investigation.

**Figure 5:**
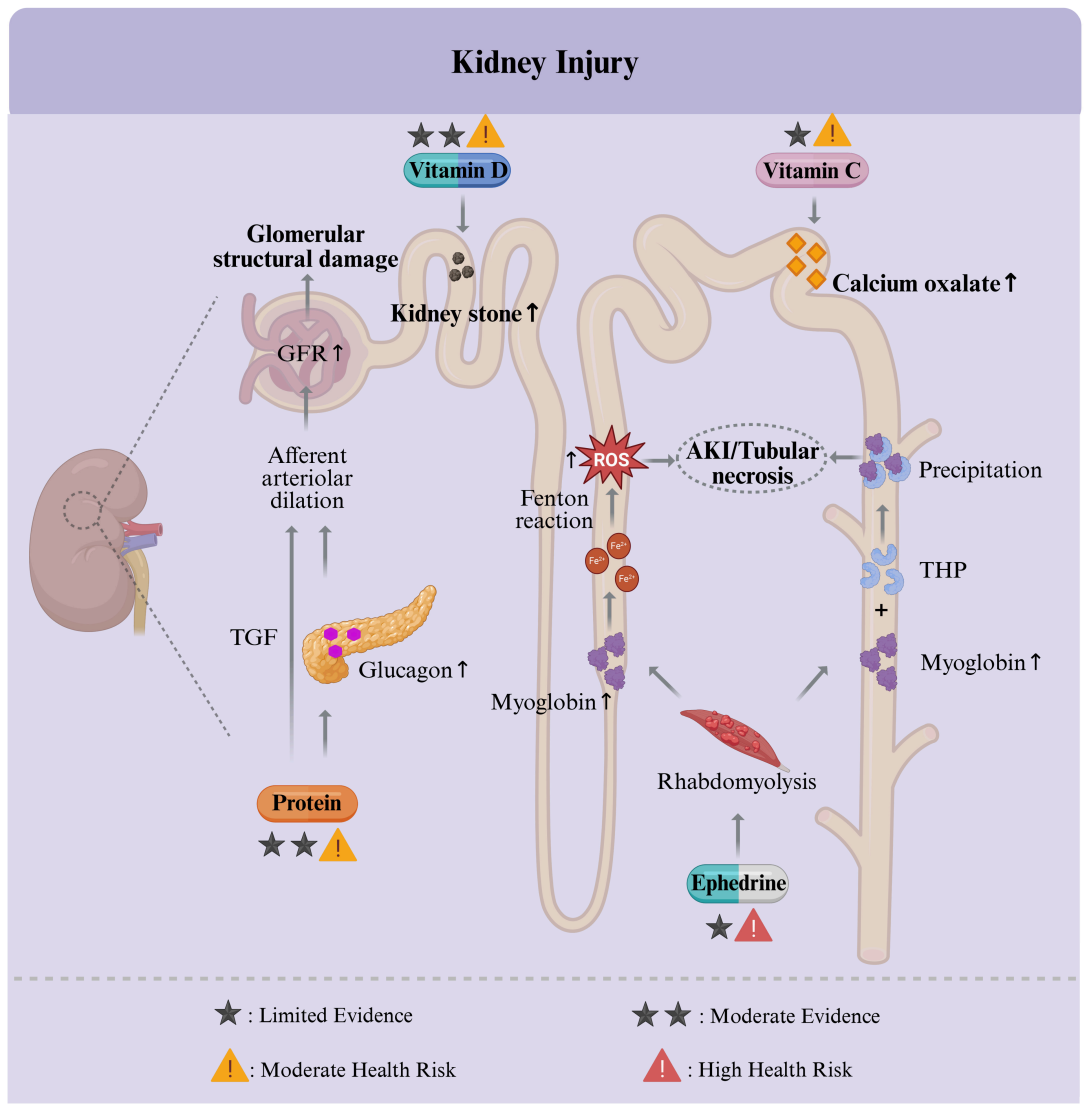
Kidney injury induced by long-term and/or high-dose sports supplement use in athletes. Long-term and/or high-dose supplementation with sports supplements, including vitamin D, vitamin C, protein, and ephedrine, in athletes may induce kidney injury, such as kidney stones, oxalate nephropathy, acute kidney injury, and renal tubular necrosis, by promoting calcium oxalate and calcium phosphate deposition, increasing GFR, and inducing rhabdomyolysis. TGF, tubuloglomerular feedback; GFR, glomerular filtration rate; THP, Tamm–Horsfall protein; Fe^2+^, ferrous ion; ROS, reactive oxygen species; AKI, acute kidney injury; ↑, increase; ↓, decrease. Created with BioRender.com.

In summary, long-term or high-dose intake of sports supplements may exert adverse effects on the kidneys through multiple mechanisms, including promoting abnormal calcium salt deposition within the kidney, increasing renal blood flow and maintaining a state of glomerular hyperfiltration, and inducing rhabdomyolysis. These processes may further lead to varying degrees of kidney injury, including kidney stones, oxalate nephropathy, AKI, and tubular necrosis (Figure 5; Table 2).

Finally, we conducted a systematic risk assessment of the potential target-organ damage associated with the aforementioned sports supplements and summarized the findings in a table to facilitate clear and intuitive interpretation. Specifically, the potential risks posed by each supplement to individual target organs were graded by comprehensively considering the likelihood or frequency of the associated adverse health effects, the severity and reversibility of the resulting damage, the duration of the adverse effects, the extent of impairment to organ function, the duration of exposure, and the vulnerability of susceptible populations. Based on this integrated assessment, the potential risks were categorized as high, moderate, or low (Table 3).

### 3.6. Supplement Contamination and Inadvertent Anti-Doping Rule Violations

In addition to their direct physiological toxicity to the endocrine and cardiovascular systems, gastrointestinal tract, liver, and kidneys, commercially available sports supplements may pose indirect risks due to inaccurate ingredient labeling or contamination during manufacturing. These risks are particularly important for competitive athletes. An international study funded by the IOC analyzed 634 nonhormonal nutritional supplements obtained from 13 countries and found that 94 products, or approximately 14.8%, contained undeclared AAS, indicating that supplement contamination is not an isolated occurrence [187]. Such contamination may result from cross-contamination during the manufacture of products containing prohibited substances, deliberate adulteration, or even the intentional misrepresentation of product ingredients [188]. Studies have shown that prohibited substances detected in supplements primarily include AAS, stimulants such as ephedrine and related compounds, and SARMs, many of which are not disclosed on product labels [106,189]. Under the World Anti-Doping Agency’s strict liability principle, athletes remain responsible for any prohibited substance detected in their samples, even when exposure results from the unintentional consumption of a contaminated supplement. Consequently, they may face serious sanctions, including periods of ineligibility from competition [190,191]. Therefore, inadvertent anti-doping rule violations resulting from supplement contamination represent one of the most important risks associated with supplement use among competitive athletes, underscoring the importance of selecting products that have undergone independent third-party testing and certification.

**Table 2:** Potential Risks of Sports Supplements.

Main Risk	Sports Supplement	Indicators	Outcomes	References
Endocrine homeostasis disruption	AAS	GnRH ↓, LH ↓, FSH ↓, Testosterone ↓, Estrogen ↓	Masculinization features, Menstrual irregularities, Testicular atrophy, Spermatogenesis disorders	[101,109,110,111]
	T_3_	TRH ↓, TSH ↓, TH ↓	Hypothyroidism, Thyroid atrophy	[100,116]
	Caffeine	CRH ↑, ACTH ↑, Cortisol ↑	Anxiety, Depression	[94,119,122]
Cardiovascular adverse effects	AAS	HDL-C ↓, LDL-C ↑	Atherosclerosis	[127,128]
	Caffeine	Sympathetic nervous system excitability ↑, Catecholamines ↑, Activation of α1 adrenergic receptors	Increased blood pressure	[134]
	Ephedrine	Sympathetic nervous system excitability ↑, Norepinephrine ↑, Activation of α_1_, β_1_, and β_2_ adrenergic receptors	Myocardial ischemia, Myocardial infarction	[104]
	2,4-DNP	Mitochondrial uncoupling in myocardium ↑	Sustained tachycardia, Acute heart failure, Sudden cardiac death	[103]
Gastrointestinal symptoms	Caffeine	Gastric mucoprotein ↓, Colonic motility ↑, Intestinal fluid secretion ↑, Catecholamines ↑	Inadequate gastrointestinal blood perfusion, Gastrointestinal barrier dysfunction	[142,143,144]
	Hypertonic carbohydrate	Intestinal lumen dilation ↑	Abdominal bloating, Diarrhea	[95]
	NaHCO_3_	CO_2_ ↑, Intestinal lumen osmotic pressure ↑	Abdominal bloating, Osmotic diarrhea, Abdominal cramping	[47]
	Fe	Gut beneficial bacteria ↓, Gut pathogenic bacteria ↑, ROS ↑	Intestinal inflammation	[96]
Hepatotoxicity	AAS	ROS ↑, Mitochondrial β-Oxidation ↑	Hepatocyte apoptosis or necrosis	[156]
	EGCG	ALT ↑, Hepatocyte mitochondrial uncoupling ↑	Hepatocyte apoptosis or necrosis	[97,160]
	SARMs	Bile salt export pump function ↓, Hepatocyte membrane permeability ↓	Cholestatic liver injury	[102]
	Creatine	Serum transaminases ↑	Hepatocyte injury	[165,166]
Kidney injury	Vitamin D	Calcium phosphate ↑, Calcium oxalate ↑	Kidney stones	[173]
	Vitamin C	Calcium oxalate ↑	Oxalate nephropathy	[175,177]
	Protein	Renal blood flow ↑, GFR ↑, Nitrogenous waste excretion ↑	Glomerular structural injury, Renal tubular metabolic burden ↑	[171,180,181,182]
	Ephedrine	Sympathetic nervous system excitability ↑, Rhabdomyolysis ↑	Acute kidney injury, Renal tubular necrosis	[184,185]

Abbreviations: AAS, anabolic–androgenic steroids; GnRH, gonadotropin-releasing hormone; LH, luteinizing hormone; FSH, follicle-stimulating hormone; T_3_, triiodothyronine; TRH, thyrotropin-releasing hormone; TSH, thyroid-stimulating hormone; TH, thyroid hormone; CRH, corticotropin-releasing hormone; ACTH, adrenocorticotropic hormone; HDL-C, high-density lipoprotein cholesterol; LDL-C, low-density lipoprotein cholesterol; 2,4-DNP, 2,4-dinitrophenol; NaHCO_3_, sodium bicarbonate; CO_2_, carbon dioxide; ROS, reactive oxygen species; EGCG, epigallocatechin gallate; ALT, alanine aminotransferase; SARMs, selective androgen receptor modulators; GFR, glomerular filtration rate; ↑, increase; ↓, decrease.

**Table 3:** Summary of the Risk Levels of Different Supplements Across Target Organs.

	Endocrine Homeostasis Disruption	Cardiovascular Adverse Effects	Gastrointestinal Symptoms	Hepatotoxicity	Kidney Injury
**AAS**	High	High	-	High	-
**T_3_ **	High	-	-	-	-
**Caffeine**	Low	Moderate	Moderate	-	-
**Ephedrine**	-	High	-	-	High
**2,4-DNP**	-	High	-	-	-
**Hypertonic carbohydrate**	-	-	Low	-	-
**NaHCO_3_**	-	-	Moderate	-	-
**Fe**	-	-	Moderate	-	-
**EGCG**	-	-	-	Moderate	-
**SARMs**	-	-	-	High	-
**Creatine**	-	-	-	Low	-
**Vitamin D**	-	-	-	-	Moderate
**Vitamin C**	-	-	-	-	Moderate
**Protein**	-	-	-	-	Moderate

Abbreviations: AAS, anabolic–androgenic steroids; T_3_, triiodothyronine; 2,4-DNP, 2,4-dinitrophenol; NaHCO_3_, sodium bicarbonate; EGCG, epigallocatechin gallate; SARMs, selective androgen receptor modulators.

## 4. Limitation

Existing studies still have several limitations. At present, human studies on the potential harms of sports supplement use in athletes are mostly based on small sample sizes, whereas large-scale prospective clinical studies remain relatively scarce. Insufficient sample sizes not only limit the identification of adverse events with low incidence but serious clinical consequences, but also make study findings more susceptible to factors such as sport type, training level, sex, and age, thereby weakening the robustness and generalizability of the current evidence. Future studies should further conduct large-sample prospective investigations with long-term follow-up to improve the quality of evidence.

In addition, although numerous studies have suggested that various sports supplements may adversely affect athletes’ health, the dose thresholds and dose–response relationships for supplement-induced adverse reactions remain incompletely defined. This not only limits the establishment of risk assessment systems, but also makes it difficult for existing studies to provide more precise guidance for sports nutrition interventions. Future research should systematically explore the dose–response relationships and risk thresholds of different sports supplements under different exercise scenarios. On this basis, risk-stratified assessment and optimization of supplementation strategies should be carried out to minimize the adverse effects of sports supplements on athletes’ health.

Meanwhile, participants in existing studies are predominantly adult male athletes, whereas insufficient attention has been paid to the potential adverse effects of sports supplement use in special athlete populations, such as adolescent athletes, female athletes, and athletes with disabilities. Because these populations differ substantially from adult male athletes in metabolic characteristics, hormonal milieu, and training adaptations, the potential harms and risk judgments derived from studies in adult male athletes cannot be directly extrapolated to these special populations. Future studies should conduct targeted research in special athlete populations to provide evidence-based support for differentiated risk assessment and individualized supplementation strategies.

## 5. Conclusions

Existing evidence suggests that long-term or high-dose intake of sports supplements may cause varying degrees of harm across multiple organ systems in athletes. These adverse effects include endocrine homeostasis disruption, cardiovascular adverse effects, gastrointestinal symptoms, hepatotoxicity, and kidney injury. Their severity may be closely associated with both the dose consumed and the duration of use. In addition, supplement use may expose athletes to the risks of product contamination and inadvertent anti-doping rule violations. Such effects may not only impair training adaptations and competitive performance but also increase the risk of acute adverse events and long-term health consequences. Safe supplementation therefore requires a coordinated, multidisciplinary approach. Clinicians and sports physicians should assess the need for supplementation based on the athlete’s health status, medication history, training load, and sport-specific demands; nutritionists should recommend only products supported by reliable evidence; dosage, duration, and relevant physiological or biochemical indicators should be regularly monitored; and coaches and athletes should prioritize products certified by reputable independent third-party organizations.

Despite growing interest in sports supplementation, the current evidence base remains limited by small sample sizes, insufficient long-term follow-up, poorly defined dose–response relationships and safety thresholds, and the predominance of adult male participants. Future research should prioritize large-scale prospective studies, clarify risk thresholds across different supplements and exercise contexts, and evaluate long-term outcomes. Greater attention should also be given to adolescent athletes, female athletes, and athletes with disabilities. Addressing these gaps will support more precise, risk-stratified, and individualized supplementation strategies while maximizing potential benefits and protecting athletes’ health and career sustainability.

## Data Availability

No new data were created or analyzed in this study. Data sharing is not applicable to this article.

## Abbreviations

The following abbreviations are used in this manuscript:

ROS	Reactive oxygen species
AIS	Australian Institute of Sport
BCAAs	Branched-chain amino acids
NaHCO_3_	Sodium bicarbonate
EGCG	Epigallocatechin-3-gallate
T_3_	Triiodothyronine
AAS	Anabolic–androgenic steroids
SARMs	Selective androgen receptor modulators
T_4_	Thyroxine
2,4-DNP	2,4-dinitrophenol
ISSN	International Society of Sports Nutrition
HPG	Hypothalamic–pituitary–gonadal
GnRH	Gonadotropin-releasing hormone
LH	Luteinizing hormone
FSH	Follicle-stimulating hormone
TRs	Thyroid hormone receptors
TRH	Thyrotropin-releasing hormone
TSH	Thyroid-stimulating hormone
THs	Thyroid hormones
HPT	Hypothalamic–pituitary–thyroid
HPA	Hypothalamic–pituitary–adrenal
CRH	Corticotropin-releasing hormone
ACTH	Adrenocorticotropic hormone
HDL-C	High-density lipoprotein cholesterol
LDL-C	Low-density lipoprotein cholesterol
VSMCs	Vascular smooth muscle cells
FDA	Food and Drug Administration
ATP	Adenosine triphosphate
I	NADH: ubiquinone oxidoreductase
II	Succinate: ubiquinone oxidoreductase
III	Ubiquinol: cytochrome c oxidoreductase
IV	Cytochrome c oxidase
ADP	Adenosine diphosphate
iFABP	Intestinal fatty acid-binding protein
HCL	Hydrochloric acid
CO_2_	Carbon dioxide
Na^+^	Sodium ion
HCO_3_^−^	Bicarbonate ion
Fe	Iron
AR	Androgen receptor
ALT	Alanine aminotransferase
NADH	Nicotinamide adenine dinucleotide
BSEP	Bile salt export pump
Ucp1	Uncoupling protein 1
H^+^	Hydrogen ion
AST	Aspartate aminotransferase
IOC	International Olympic Committee
Calcitriol	1,25-dihydroxyvitamin D
DHA	Dehydroascorbic acid
2,3-DKG	2,3-diketogulonic acid
NaCl	Sodium chloride
TGF	Tubuloglomerular feedback
GFR	Glomerular filtration rate
THP	Tamm–Horsfall protein
AKI	Acute kidney injury
Fe^2+^	Ferrous ion
ULs	Tolerable upper intake levels
GRADE	Grading of Recommendations Assessment, Development and Evaluation
GI	Gastrointestinal
FFM	Fat-free mass
DILI	Drug-induced liver injury
IGF-1	Insulin-like growth factor 1
